# Sodium butyrate exerts a neuroprotective effect in rats with acute carbon monoxide poisoning by activating autophagy through the mTOR signaling pathway

**DOI:** 10.1038/s41598-024-55198-z

**Published:** 2024-02-26

**Authors:** Jing Wen, Qiong Xu, Jing Li, Xuanyang Shen, Xiaolong Zhou, Jing Huang, Shiping Liu

**Affiliations:** 1 Department of Emergency, Affiliated Hospital of North Sichuan Medical College, Nanchong, 637000 China; 2 Department of General Medicine, Affiliated Hospital of North Sichuan Medical College, Nanchong, 637000 China; 3 North Sichuan Medical College Innovation Center for Science and Technology, Nanchong, 637000 China

**Keywords:** Neuroscience, Medical research, Neurology, Pathogenesis

## Abstract

Acute carbon monoxide (CO) poisoning is a prevalent type of poisoning that causes significant harm globally. Delayed encephalopathy after acute carbon monoxide poisoning (DEACMP) is a severe complication that occurs after acute CO poisoning; however, the exact underlying pathological cause of DEACMP remains unclear. Accumulating evidence indicates that abnormal inflammation and immune-mediated brain damage, cellular apoptosis and autophagy, and direct neuronal toxicity are involved in the development of delayed neurologic sequelae. Sodium butyrate, a histone deacetylase inhibitor, has gained increasing attention for its numerous beneficial effects on various diseases, such as obesity, diabetes, inflammatory diseases, and cerebral damage. In this study, an acute carbon monoxide poisoning (ACOP) model is established in rats to investigate the mechanism of CO poisoning and the therapeutic potential of sodium butyrate. The results suggested that the ACOP rats had impaired spatial memory, and cell apoptosis was observed in the hippocampi with activated autophagy. Sodium butyrate treatment further increased the activation of autophagy in the hippocampi of CO-exposed rats, inhibited apoptosis, and consolidated spatial memory. These findings indicated that sodium butyrate may improve memory and cognitive function in ACMP rats by promoting autophagy and inhibiting apoptosis.

## Introduction

Acute carbon monoxide poisoning (ACOP) is one of the most common clinical poisonous diseases as well as the primary fatal cause of accidental poisoning worldwide^1^. Delayed neurologic sequelae are the most serious complications associated with acute CO poisoning. Despite receiving treatment, approximately 0.2–40% of survivors develop delayed encephalopathy after acute carbon monoxide poisoning (DEACMP)^2^.DEACMP is characterized by a series of neurological and psychiatric symptoms, and the clinical manifestations of DEACMP include dementia, poor concentration, memory loss, cognitive and personality changes, and dysfunction of the pyramidal and extrapyramidal systems^3^. The pathogenic mechanisms of DEACMP include the ischemia-hypoxia hypothesis, the inflammation and immune-mediated damage hypothesis, and mechanisms associated with cellular apoptosis and direct neuronal toxicity induced by CO^4^. However, these hypotheses have not been able to provide a satisfactory explanation for the complex clinical presentation of DEACMP.

Autophagy is a catabolic process used to degrade and recycle the cell’s own unnecessary or dysfunctional components through the lysosomal system. Normally, it facilitates the cell to maintain homeostasis, prevents the accumulation of toxic or damaging proteins and organelles, and maintains the function of cellular physiology^5^.Autophagy increases significantly in response to various stimuli, including nutrient starvation, growth factor deprivation, DNA damage, the accumulation of abnormal proteins, and organelle damage^6^.Under pathological conditions of the cell, the regulation of autophagy facilitates better adaptation to stresses such as starvation, hypoxia, and ischemia^7^. In recent years, emerging evidence has suggested that autophagy is closely linked to neurological disorders, and the activation and upregulation of autophagy improves neurological deficits that play vital roles in neuroprotection^8,9^. It has been reported that the upregulation of autophagy alleviates the deposition of pathologic proteins (Aβ and tau protein) and improves learning and memory in perioperative neurocognitive disorders in rats with diabetes^10^, It also suppresses neuronal apoptosis in Parkinson's model rats. In addition, activation of autophagy is beneficial to reducing neurological injury and improving the prognosis of ischemic brain damage; however, few studies have focused on autophagy in DEACMP.

Sodium butyrate is a histone deacetylation inhibitor (HDACi) that is produced in the colon and primarily originates undigested dietary carbohydrates by intestinal bacterial fermentation^11^. It is of particular interest because of its multiple biological functions, including anti-inflammatory, anticancer, immune modulation, regulation of intestinal flora, and maintenance of gastrointestinal tract mucosal integrity^12,13^. It has been shown that sodium butyrate prevents hypobaric hypoxia-induced spatial memory deficits^14^. In an animal model of posttraumatic stress disorder, sodium butyrate reversed single prolonged stress-induced hippocampal histone deacetylase (HDAC) overexpression and enhanced fear elimination^15^. Further, sodium butyrate improves synaptic plasticity by reducing neuroinflammation in 5XFAD mice early in the disease^16^.

The therapeutic effects and mechanisms of sodium butyrate in CO-induced brain injury remain unclear. In this study, we established an ACOP model in rats. We found that the ACOP rats exhibited a loss of spatial memory, increased apoptosis, and activated autophagy in the hippocampus. Sodium butyrate treatment further promoted autophagy, inhibited apoptosis, and facilitated the retention of spatial memory in ACOP rats.

## Results

### Manifestations of acute CO poisoning

After exposure to CO for 5–10 min, the rats showed excitement and mild agitation, followed by shortness of breath, increased respiratory amplitude, cherry-red coloration of the mucous membranes of the mouth and nose, and cherry-red coloration of the skin of the ears, paws, and tail. Some rats showed erect hair, mania, and limb paralysis, and some rats had convulsions and opisthotonos. When the CO concentration was maintained at 3000 ppm, the rats gradually lost consciousness or even died. Autopsy of the dead rats showed severely edematous brain tissue and significant internal organ hyperemia. After being removed from the container, the rats gradually regained consciousness after inhaling fresh air for 10–15 min. The rats in the control group had smooth respiration, normal skin mucosa, no irritability, no convulsions, and no congestion and edema in the internal organs of the dissected rats. The dead rats were removed from the experiment, and the same number of new rats were trained using the Morris water maze (MWM) test. A total of 16 rats died during the model establishment, and no rats died 14 days after CO exposure.

### Carboxyhemoglobin levels following CO exposure

**Figure 1:**
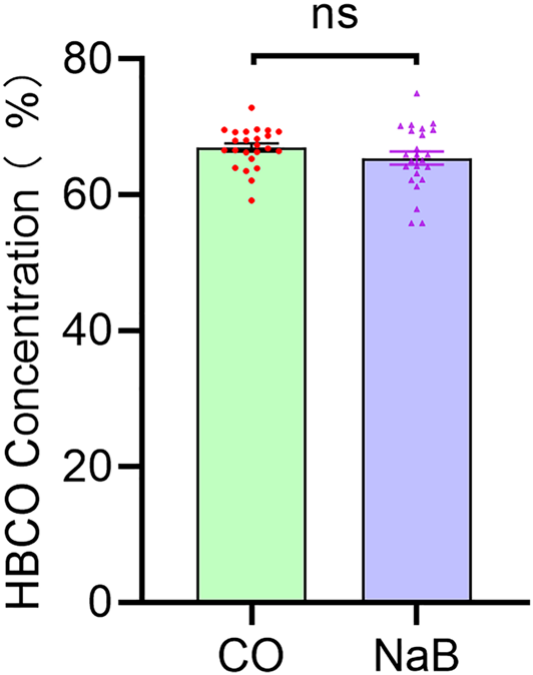
Carboxyhemoglobin levels of rats after CO exposure. After exposure to CO for 60 min, the mean blood carboxyhemoglobin concentration reached toxic levels in the CO group (66.94 ± 2.923) and NaB group (65.38 ± 4.747), there was no difference between the two groups.

Rats with a blood carboxyhemoglobin concentration at or above 50% after CO poisoning, loss of consciousness, and recovery of spontaneous respiration after resuscitation were selected for the experiment. The concentrations of carboxyhemoglobin in the blood are shown in Fig. 1.

### Sodium butyrate treatment attenuated CO-induced spatial memory impairment

**Figure 2:**
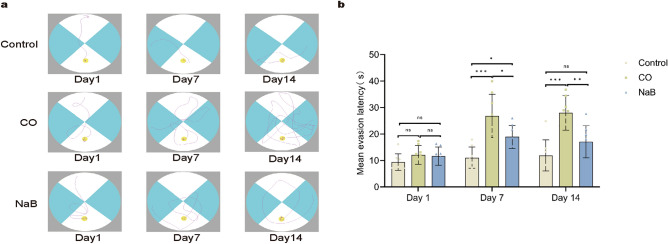
sodium butyrate treatment consolidated spatial memory in the rats after CO exposure. (**a**) Morris water maze track diagram for each group. The representative motion track from single animals in the CO group was generally more complex than that in the control group. The representative motion track from single animals of the NaB group was simpler than that in the CO group. (**b**) Mean escape latency time for each group, the original images are presented in Fig. S13. Data are presented as mean ± SD (n = 8). ns: no significant, *p < 0.05, **p < 0.01, ***p < 0.001. *CO* carbon monoxide poisoning, *NaB* sodium butyrate.

We evaluated the effect of sodium butyrate on the CO-induced memory and cognitive dysfunction via the Morris water maze (MWM) test. The data showed that the poisoning rats exhibited a significantly prolonged platform identification latency compared with normal ones on days seven and 14 (Fig. 2a,b). However, sodium butyrate treatment alleviated the loss of spatial memory function in the CO-exposed rats, and significantly shortened escape latency were observed in the NaB group compared with those in the CO group on days seven and 14 (Fig. 2a,b). There was no statistical difference in the swimming latency between the three experimental groups on day one (Fig. 2a,b). The representative swimming trials are shown in Fig. 2a. These results suggested that spatial memory was impaired in the ACOP rats compared with the normal rats, but the sodium butyrate treatment consolidated spatial memory in the rats after CO exposure.

### Sodium butyrate reduced the expression of HDAC1 in rats after CO exposure

**Figure 3:**
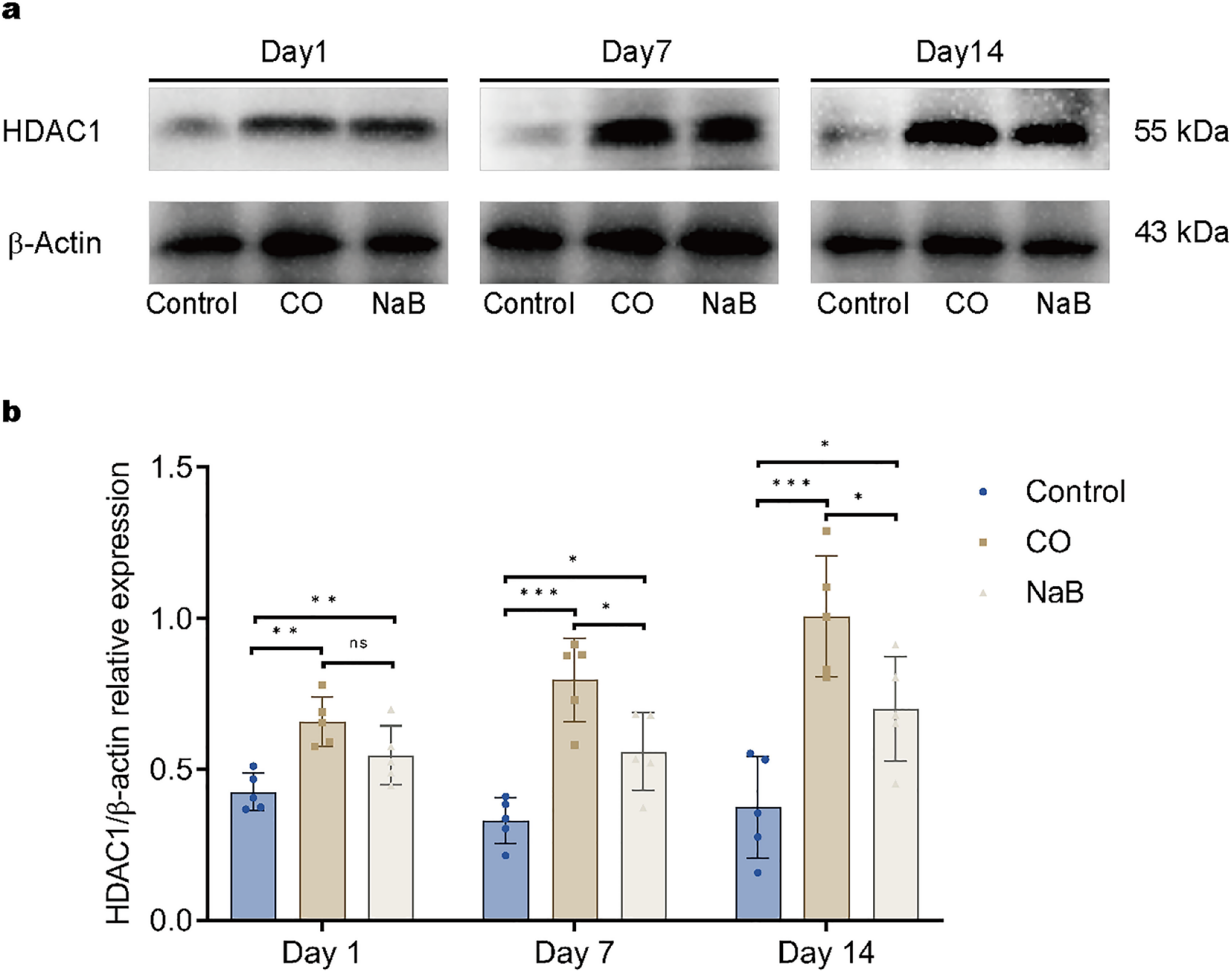
Sodium butyrate inhibited CO-induced HDAC1 protein overexpression in rat hippocampal tissue. (**a**) Representative images of Western blots for hippocampal HDAC1 on day 1, day 7, and day 14 after exposure to CO. These blots was cropped, the original, un-processed blots are presented in Fig. S8, 9. (**b**) Quantitative analysis of Western blot for HDAC1. Protein levels of HDAC1 are normalized to the level of β-actin. Data are expressed as mean ± SD (n = 5/group). ns: no significant, *p < 0.05, **p < 0.01, and ***p < 0.001. *CO* carbon monoxide poisoning, *NaB* sodium butyrate.

It has been reported that the expression of the HDAC1 protein is increased in hypoxia and ischemia/reperfusion models, and HDAC1 overexpression decreases cell survival, whereas inhibition or knockdown of HDAC1 increases cell viability^17,18^. Therefore, we investigated the effect of sodium butyrate on HDAC1 after CO poisoning. Western blotting was performed to evaluate the expression of HDAC1 protein on day one, day seven, and day 14 after CO exposure. Our results demonstrated that there was a marked upregulation in the protein level of HDAC1 in the CO group compared with the control group at all of the time points (Fig. 3a,b). With prolonged poisoning, the HDAC1 protein expression gradually increased. Conversely, sodium butyrate treatment decreased the HDAC1 protein level in the CO-exposed rats (Fig. 3a,b). There was no significant difference between the NaB group and the CO group on day one (Fig. 3b). However, a significant decrease in the HDAC1 protein levels was observed in CO-poisoned rats on days seven and 14 after sodium butyrate treatment (Fig. 3b). These data suggested that sodium butyrate inhibited CO-induced HDAC1 protein overexpression in rat hippocampal tissue.

### Sodium butyrate increased the expression of the autophagy-related proteins and reduced apoptosis protein expression

**Figure 4:**
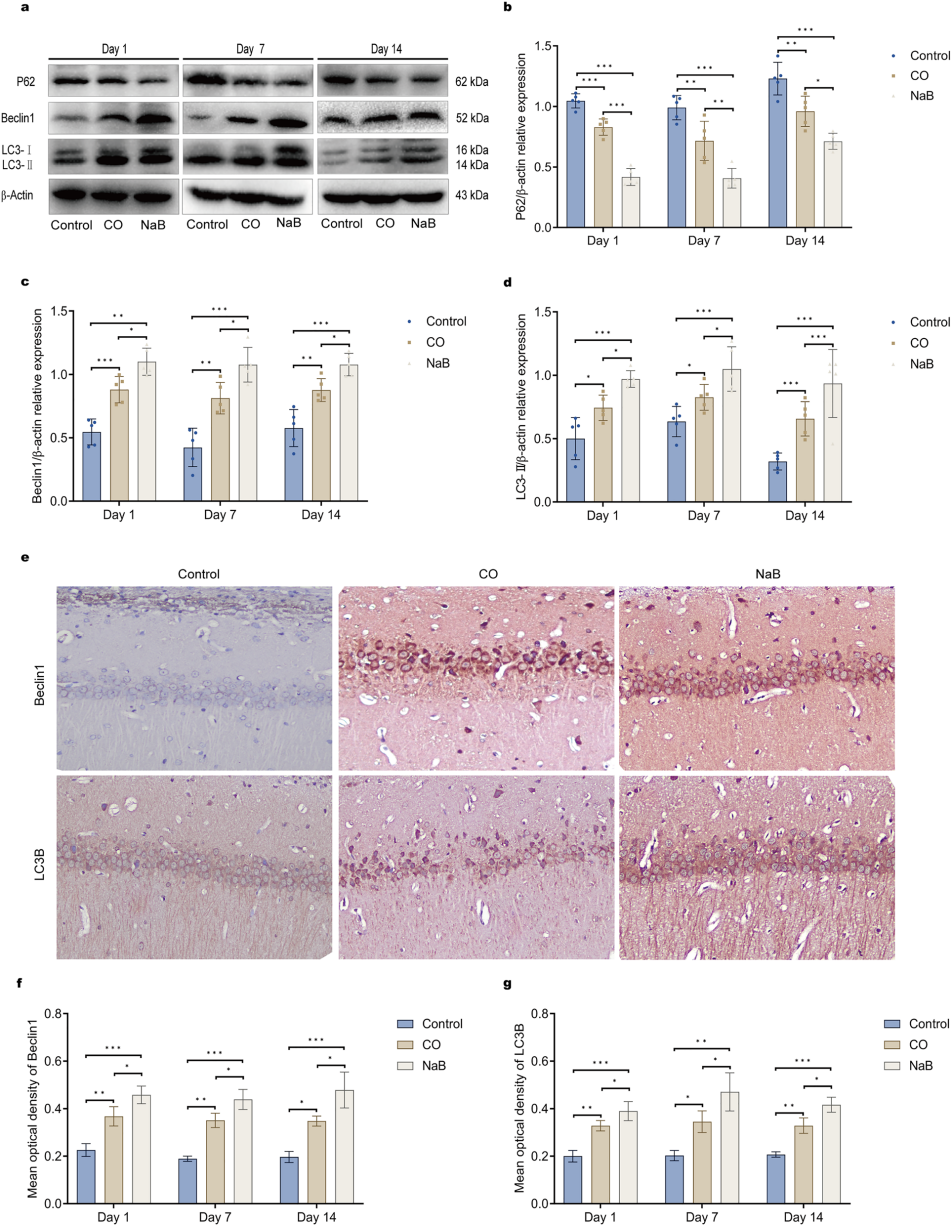
Sodium butyrate treatment promotes the expression of autophagy-related proteins in ACOP rats. CO poisoning can increase autophagy activity of rat hippocampal neuronal cells, CO upregulated the expressions of Beclin1 and LC3II, and sodium butyrate treatment further promoted autophagy. (**a**) Representative images of Western blots for hippocampal P62, Beclin1, and LC3-B on day1, day7, and day14 after exposure to CO; These blots was cropped, the original, un-processed blots are presented in Fig. S10a,b,c. (**b–d**) Quantitative analysis of Western blot for P62,Beclin1,and LC3-B.The protein levels of P62, Beclin1, and LC3-II are normalized to the level of β-actin, data are expressed as mean ± SD (n = 5/group); **(e)** Immunohistochemical staining for Beclin1 and LC3B in the CA1 area of hippocampi on day14 after exposure to CO, the detection of Beclin1 and LC3B, appearing as yellow–brown, brown, and deep brown. Compared with the control group, Beclin1 and LC3B immunopositivity in the nerve fibers of the CO group was significantly increased, and a wider and longer distribution of Beclin1 and LC3B immunopositivity was observed in the NaB group, Scale bars = 100 μm; the original images are presented in Fig. S16a–b,c, 18a–b,c. (**f**–**g**) Quantitative results for Beclin1 and LC3B (n = 3/group). ns: no significant, *p < 0.05, **p < 0.01, and ***p < 0.001. *CO* carbon monoxide poisoning, *NaB* sodium butyrate.

Prior studies have reported that autophagy, as a degradation/recycling system, is associated with the pathogenesis of many neurological disorders. We examined the expression of autophagy-related proteins in order to investigate the relationship between autophagy and neurologic injury after CO poisoning and the therapeutic effects of sodium butyrate. A western blot assay showed that CO significantly upregulated the expression of autophagy-related proteins Beclin1 and LC3II and inhibited P62 expression at all of the time points, indicating that CO poisoning can increase autophagy activity of rat hippocampal neuronal cells (Fig. 4a–d). However, the expressions of Beclin1 and LC3II were further increased, and that of P62 was further decreased in the hippocampi of rats from the NaB group, suggesting that sodium butyrate treatment further promoted autophagy (Fig. 4a–d). Immunohistochemical staining results of the autophagy-related proteins, Beclin1 and LC3B, in the brain tissues of rats from all of the groups were consistent with the western blot results, and the immunopositivity rates of Beclin1 and LC3B were elevated at all of the time points in the CO group (Fig. 4e–g and S1, 2). After sodium butyrate treatment, the immunopositivities of Beclin1 and LC3B were further elevated (Fig. 4e–g and S1, 2), and they were more densely and widely distributed (Fig. 4e and S1, 2).

**Figure 5:**
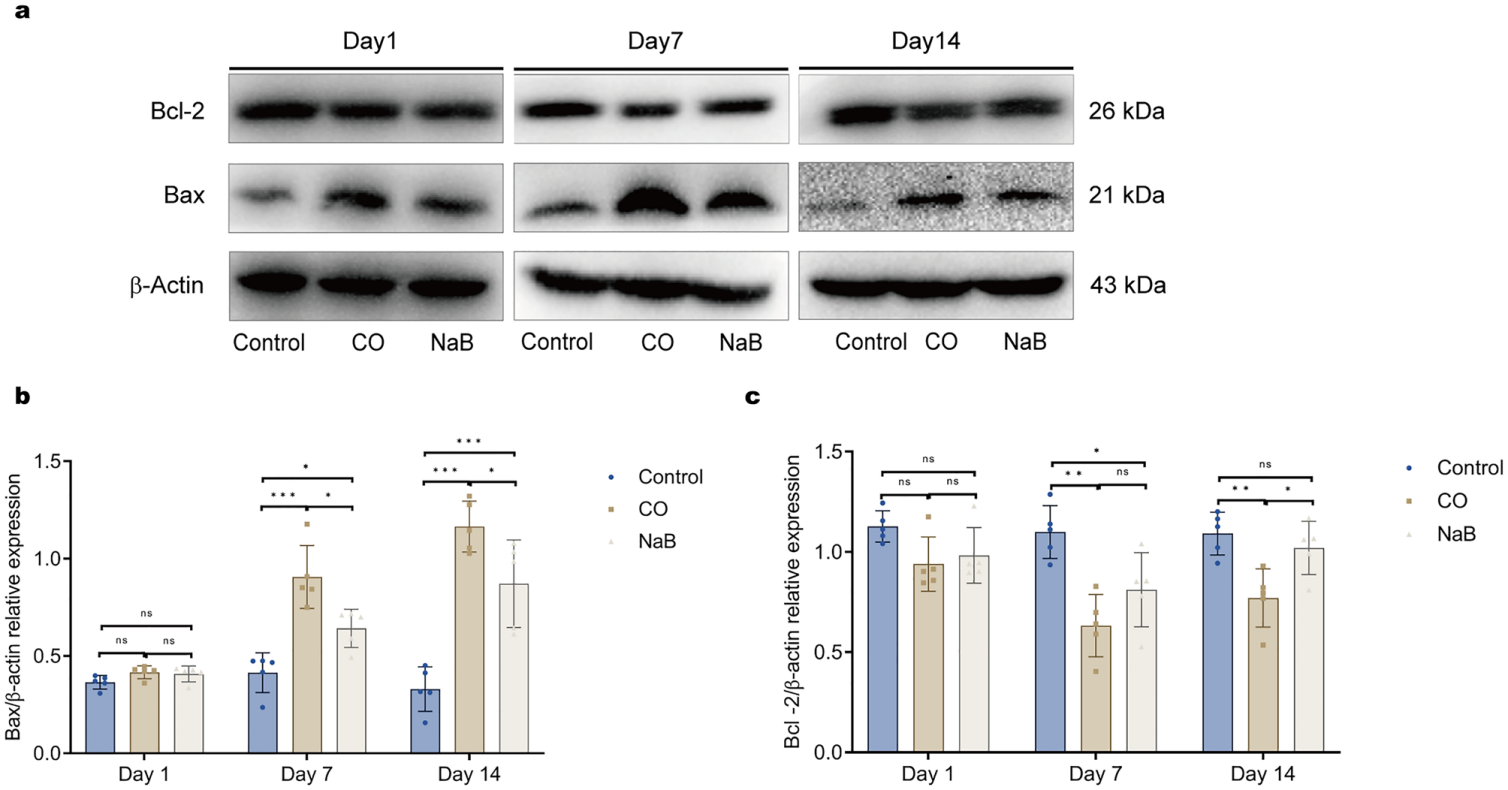
Sodium butyrate inhibits cell apoptosis in the hippocampi. In the CO group, the expression of Bax gradually increased, accompanied by a decrease in Bcl-2, sodium butyrate treatment reversed this trend. (**a**) Representative images of Western blots for hippocampal Bax and Bcl-2 on day1, day7, and day14 after exposure to CO; These blots was cropped, the original, un-processed blots are presented in Fig. S11a–b,c. (**b**–**c**) Quantitative analysis of Western blot for Bax and Bcl-2. Protein levels of Bax and Bcl-2 are normalized to the level of β-actin. Data are expressed as mean ± SD (n = 5/group). ns: no significant, *p < 0.05, **p < 0.01, and ***p < 0.001. *CO* carbon monoxide poisoning, *NaB* sodium butyrate.

Therefore, autophagy is closely associated with apoptosis. We also examined the apoptosis-related proteins, Bcl-2 and Bax. The results showed that the Bax expression gradually increased in the hippocampi of the CO group rats, accompanied by a decrease in Bcl-2 (Fig. 5a,b,c). Sodium butyrate reversed this trend (Fig. 5a,b,c). These data suggested that carbon monoxide activated autophagy and apoptosis, and the intervention of sodium butyrate further promoted autophagy and inhibited apoptosis.

### Sodium butyrate inhibited the mTOR signaling pathway

**Figure 6:**
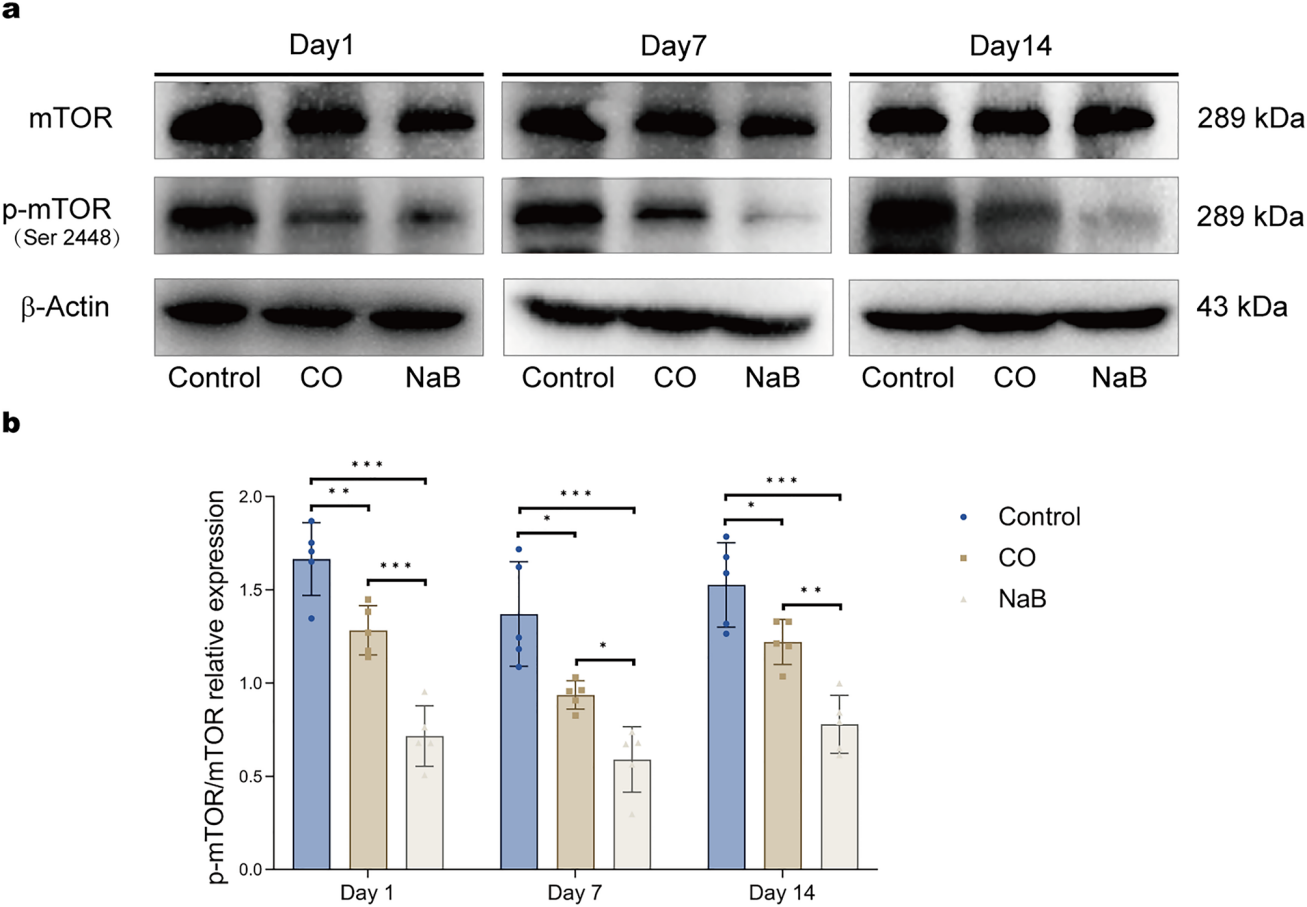
Sodium butyrate inhibited the mTOR signaling pathway. Western blot analysis for assessing the effects of sodium butyrate on the phosphorylation of mTOR signaling in the hippocampus of rats after CO exposure. (**a**) Representative images of Western blots for hippocampal mTOR and p-mTOR.These blots was cropped, the original, un-processed blots are presented in Fig. S12a–b,c. (**b**) Quantitative analysis of Western blot for the expression of p-mTOR and p-mTOR, the level of p-mTOR was normalized to the level of mTOR. Data are expressed as mean ± SD (n = 5/group). *p < 0.05, **p < 0.01, and ***p < 0.001. *CO* carbon monoxide poisoning, *NaB* sodium butyrate.

The mammalian target protein of rapamycin (mTOR) is a key molecule that regulates autophagy. We then investigated the expression of mTOR. We found that the ratio of p-mTOR/mTOR in the hippocampi of rats in the CO group was significantly decreased at all of the time points compared with those of the control group (Fig. 6a,b). After the sodium butyrate treatment, the expression of p-mTOR/mTOR was lower than that of the model rats, and the difference was statistically significant (Fig. 6a,b).

### Pathological changes in the hippocampi of the rats in the three groups

#### Hematoxylin and eosin staining

**Figure 7:**
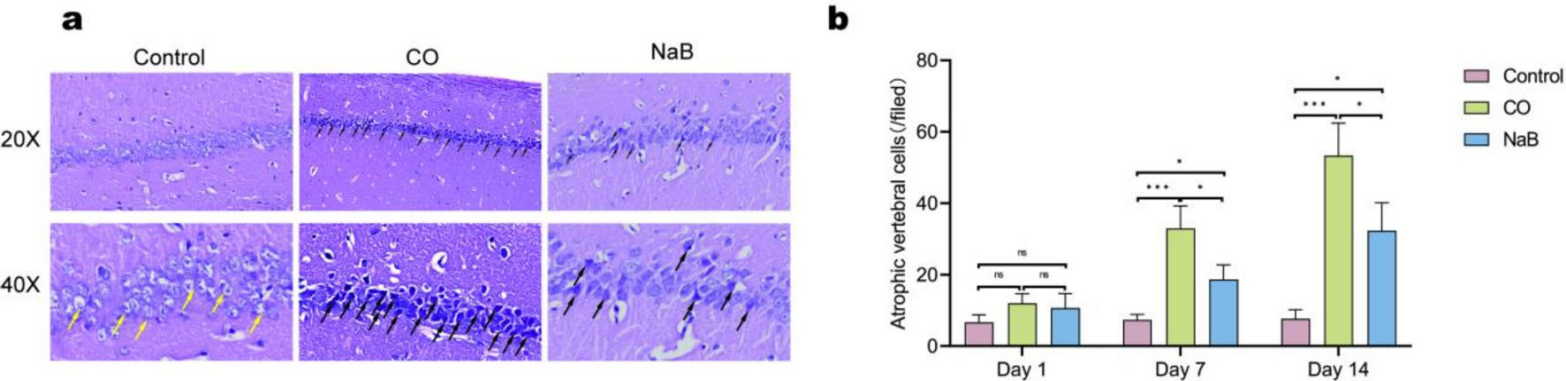
Sodium butyrate treatment alleviated the neuronal damage of the hippocampi in rats following CO exposure. (**a**) Representative graphs of the pathologic changes in the CA1 region of the hippocampi on 14 days after CO exposure. Yellow arrows are normal neurons, with round or oval shapes, intact nuclear membranes, clear outlines, lightly stained nuclei, black arrows point out the damage cells, atrophic vertebral cells, they were irregular shapes and disorganized cytosolic structures, the nucleoli appeared deeply stained and deviated., Scale bar = 100 μm and Scale bar = 50 μm;the original images are presented in Fig. S14a,b,c (**b**) Quantitative analysis of atrophic vertebral cells, (n = 3/group).The cell count results are expressed as cells/field (200× magnification).ns: no significant, *p < 0.05, **p < 0.01, and ***p < 0.001. *CO* carbon monoxide poisoning, *NaB* sodium butyrate.

Hematoxylin and eosin (H&E) staining was performed to observe the neurons in the hippocampal area on one, seven, and 14 days after ACOP model induction. We found that neurons in the CA1 region of the hippocampal tissue in the control groups rats were neatly and tightly arranged, with round or oval shapes, intact nuclear membranes, clear outlines, lightly stained nuclei, and no obvious changes at all of the time points (Fig. 7a,b and S3). However, in the CO group, neurons in the CA1 regions showed obvious pathological changes, with irregular shapes and disorganized cytosolic structures. In addition, the nucleoli appeared deeply stained and deviated, part of which was accompanied by pyknosis and karyorrhexis. The number of cells was reduced, the cell layer was thinned, and the most obvious cellular damage was observed at 14 days of toxicity (Fig. 7a,b and S3). While, the cellular damage in rats in the NaB groups was reduced significantly compared with that of the CO groups, the neuronal bodies returned to roughly normal dimensions, and fewer nuclear pyknosis and karyorrhexis were detected (Fig. 7a,b and S3). This result suggested that sodium butyrate treatment alleviated the neuronal damage of the hippocampi in rats following CO exposure.

#### Nissl staining

**Figure 8:**
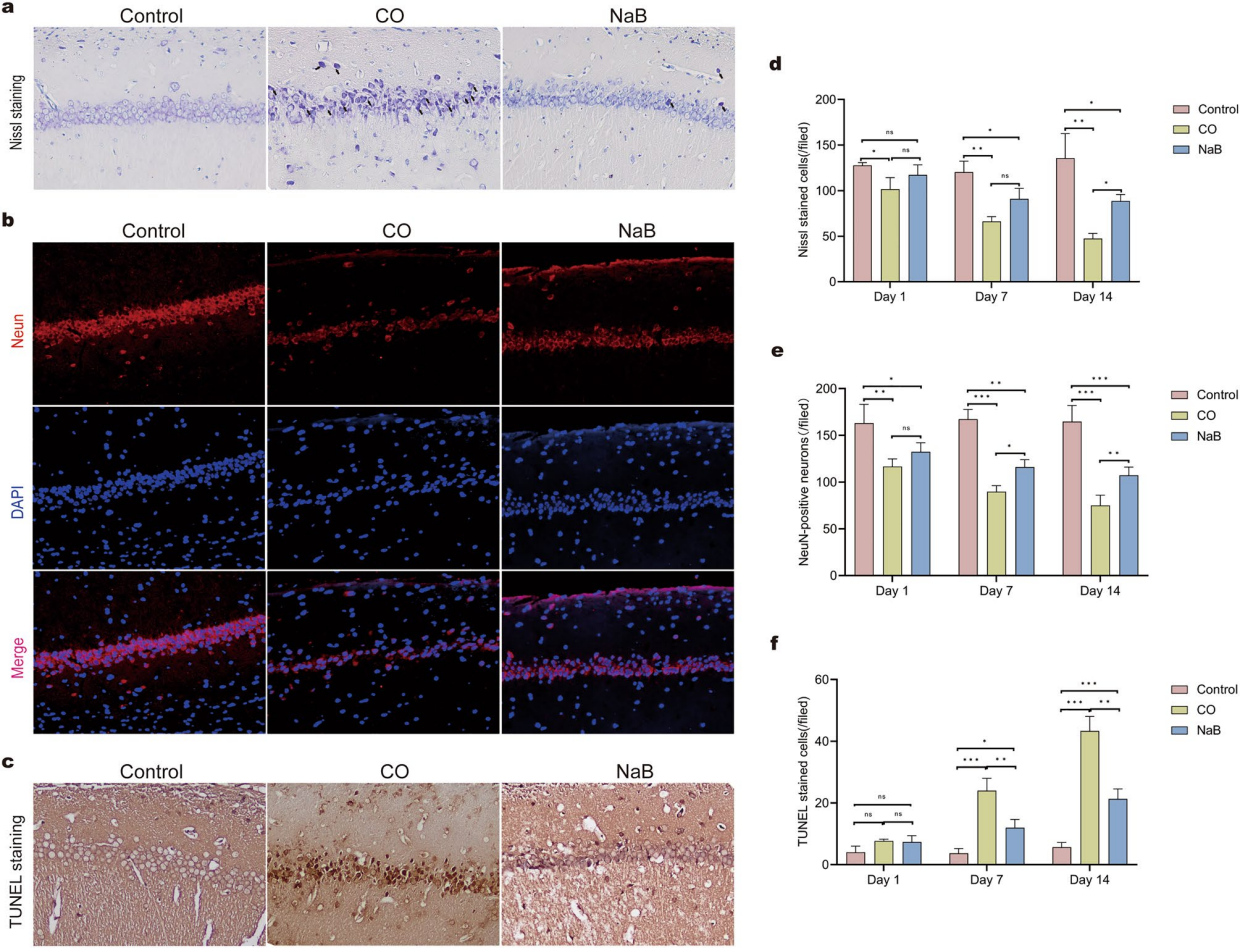
Sodium butyrate reduced neuronal death and apoptosis induced by CO poisoning**.** Rat brains were removed on 1,7, and 14 days after CO exposure and subjected to Nissl, NeuN staining and TUNEL staining, these images show the pathologic changes on 14 days after CO poisoning; (**a**) Representative graphs of Nissl staining in the CA1 region of the hippocampi, black arrows in the picture indicated pyknosis Nissl bodies. Scale bars = 100 μm; the original images are presented in Fig. S17a–b,c. (**b**) Representative graphs of NeuN (red) staining in theCA1 region of the hippocampi. Scale bars = 100 μm; the original images are presented in Fig. S15a,b,c. (**c**) Representative graphs of TUNEL staining in theCA1 region of the hippocampi, apoptotic cells were stained a deep brown color, Scale bars = 100 μm. the original images are presented in Fig. S19a–b,c. (**d**–**f**) Respective statistical analysis of Nissl-stained cells, NeuN-positive neurons, and TUNEL-positive cells, (n = 3/group). The cell count results are expressed as cells/field (200× magnification). ns: no significant, *p < 0.05, **p < 0.01, and ***p < 0.001. *CO* carbon monoxide poisoning, *NaB* sodium butyrate.

Nissl staining was performed on the CA1 region of the hippocampal neurodegeneration 14 days after ACOP model induction in order to investigate the effect of sodium butyrate on CO-induced hippocampal neurodegeneration. We found a significant reduction in the number of hippocampal neurons in rats after CO exposure, the shrinkage or detachment of neuronal cell bodies, and significant reductions or losses of Nissl bodies compared with rats in the control group (Fig. 8a and S4). In the NaB group, the number of hippocampal neurons increased significantly compared with those in the CO group, thus suggesting that neuronal damage in the hippocampus after CO poisoning was alleviated by sodium butyrate intervention. Similar data were observed when the numbers of Nissl-positive cells were quantified (Fig. 8d and S4).

#### Immunochemical and TUNEL Staining

We analyzed the results of the terminal deoxynucleotidyl transferase dUTP nick end labeling (TUNEL) assay and NeuN staining in the CA1 area of the hippocampus on day 14 after CO exposure in order to further confirm the neuroprotective effect of sodium butyrate. The data showed a significant decrease in the number of NeuN-positive surviving neurons accompanied by an increase in apoptotic cells in the CO group compared with the control group. Sodium butyrate treatment limited the increase in apoptotic cells and augmented the number of NeuN-positive neurons, indicating an anti-apoptotic action of sodium butyrate (Fig. 8b,c,e,f and S5, 6).

#### Transmission electron microscopy (TEM)

**Figure 9:**
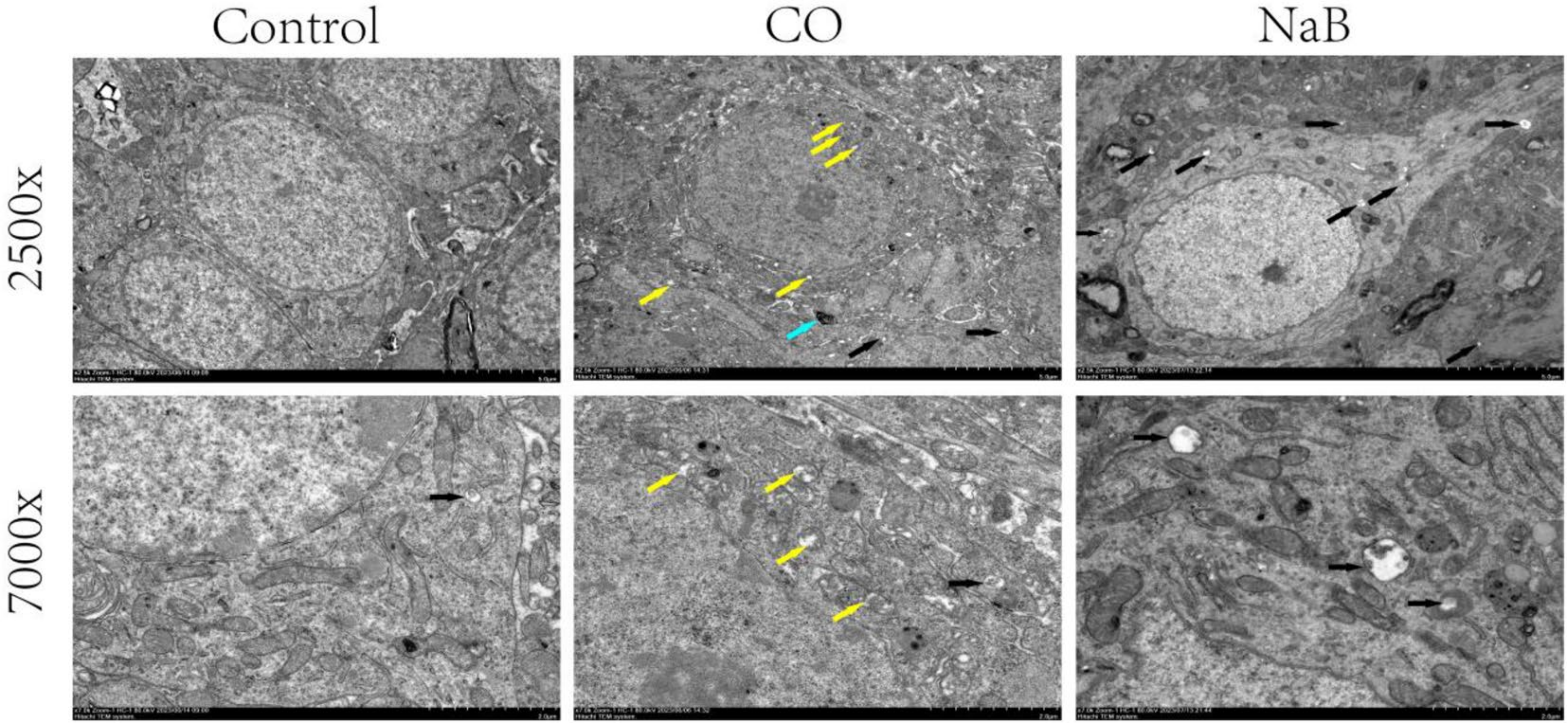
Transmission Electron Microscopy observation of ultrastructural of brain hippocampal nerve cells in three groups. The figure shows the ultrastructure of neuronal cells in the hippocampal region of the rat on 14 days after CO poisoning. Black arrows are autophagosomes, yellow arrows are edematous mitochondria, blue arrows are demyelination. Scale bar = 5 μm and Scale bar = 2 μm;the original images are presented in Fig. S20.

In the prior section, we found increased expression of autophagy-related proteins in the ACOP rats. In this portion, autophagosomes were investigated using TEM. The cell morphologies in the hippocampi of rats in the control group were normal and structurally intact, with big and round nuclei, uniform chromatins, and few autophagosomes were observed. In the CO group, the observation of cell morphologies showed abnormal mitochondrial morphologies, including obvious mitochondrial swelling, mitochondrial cristae ruptures, and mitochondrial vacuolization. In addition, autophagic vesicles appeared in the cytoplasms, and partial demyelination of neurons was observed (Fig. 9). The degree of damage of the hippocampal ultrastructure in the NaB group was a little less than that of the CO group, the mitochondria were normal or only slightly swollen with few vacuoles, and the number of autophagosomes in the hippocampal neurons was increased compared with that of the CO group (Fig. 9). The results suggested that sodium butyrate treatment efficiently improved the ultrastructural damage of the hippocampi induced by CO poisoning.

## Discussion

DEACMP is a common and serious complication of CO poisoning. It has been reported that^19^, the lethality rate of patients with DEACMP is approximately 30%, and the disability rate is approximately 78%. Once DEACMP occurs, it will lead to a significant decline in the quality of life and cause a heavy economic burden on patients, their families, and society. As a key part of learning and memory, the hippocampus plays an important role in memory shaping and spatial localization^20^, and it is a region of the brain that is most sensitive to ischemic-hypoxic injury^21^. The Morris water maze test, a vital tool for assessing cognitive function in rats, is a key technique in the investigation of hippocampal function^22^. In this study, rats were trained to eliminate those with poor learning and memory abilities before the experiment, thus minimizing the effect of individual differences and improving the reliability of the results. This work explored the mechanism of brain damage after CO exposure by establishing an ACOP rat model. The results indicated that the ACOP rats experienced impaired spatial memory, increased apoptosis in the hippocampi, and activated autophagy.

The development of DEACMP is closely related to brain cell apoptosis^23^. After carbon monoxide poisoning, the formation of COHb and the direct toxicity of CO lead to tissue hypoxia. Tissue hypoxia-induced oxidative stress and the neuroinflammatory response persists a long time after CO exposure, which induces apoptosis and necrosis of brain cells, ultimately leading to a wide range of neuropathological and physiological alterations^24,25^. In this study, we observed and analyzed the data on days one, seven, and 14 after CO poisoning. The results showed that the expressions of the apoptotic protein, Bax, gradually increased in the hippocampi of the ACMP rats and the expressions of the anti-apoptotic protein, Bcl-2, gradually decreased compared with that of the normal rats. During the early stage of poisoning, the degree of neuronal damage was mild, the number of surviving neurons was high, and the number of apoptotic cells was low. With prolongation of the disease, the neuronal cell damage gradually increased, and the cell morphology changed significantly, with a decrease in the number of NeuN-positive neurons and an increase number of apoptotic cells. In addition, there was a disorganized arrangement of the cell layers. The Morris water maze test showed that the mean swimming avoidance latency of the poisoned rats was significantly prolonged from 7 to 14 days after intoxication, suggesting that the spatial memory was impaired by CO exposure in the rats. These results were consistent with previous findings that reflected a delay in brain damage after CO exposure. However, we observed a significant shortening of the Morris water maze latency, a decrease in the Bax protein levels, an increase in the number of surviving neurons, and a decrease in the number of apoptotic cells in the hippocampal CA1 region of the sodium butyrate-treated rats. H&E staining and Nissl staining showed a decrease in the apoptotic and necrotic cells in the ACMP rats treated with sodium butyrate, and the disappearance of Nissl bodies was observed only in a few neurons. These results suggested that the sodium butyrate intervention ameliorated CO-induced brain damage and consolidated memory in the ACOP rats.

**Figure 10:**
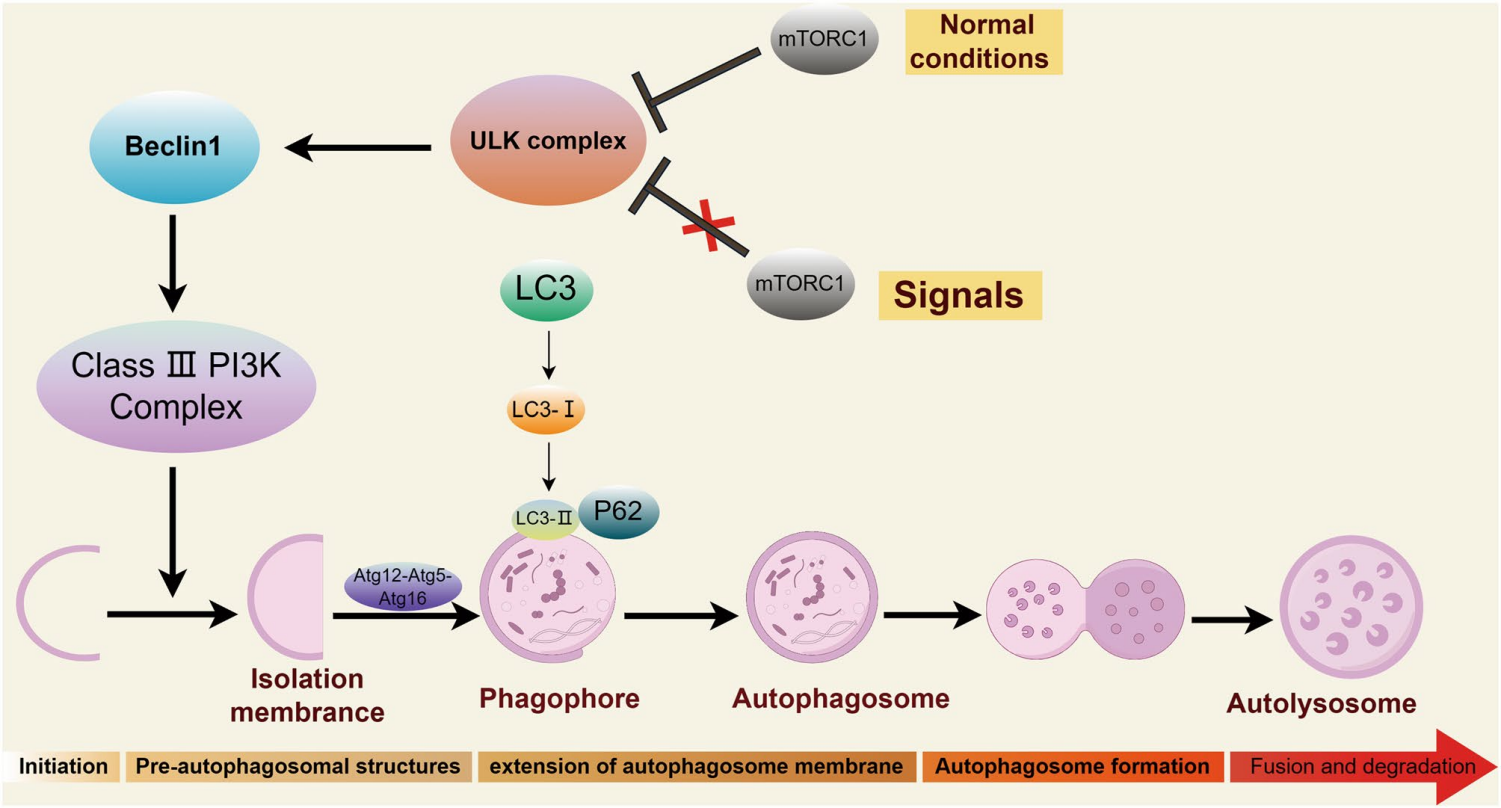
The mechanism of autophagy. (By Figdraw.)

Autophagy plays a vital role in maintaining the dynamic balance of body tissues and is closely linked to apoptosis and necrosis. It is also closely associated with neurological diseases. Hypoxia, a high generation of reactive oxygen species, and a reduced cellular energy supply are all critical factors for the initiation of autophagy^26,27^. The ULK1 (unc-51-like kinase 1) complex, consisting of ULK1, ULK2, Atg (autophagy-associated protein) 13, Atg 101, and FIP200 (focal adhesion kinase family interacting protein of 200 kDa), is critical for autophagy initiation^28^. mTOR is involved in the formation of two distinct complexes, mTORC1 and mTORC2^29^. Under normal growth conditions, mTORC1 induces hyperphosphorylation of Atg13 and ULK1/2 that inhibits the kinase activity of ULK, leading to down-regulation of autophagy in mammalian cells^29–31^. Hypoxia can lead to dephosphorylation of mTOR at serine^32^, resulting in the inactivation of mTORC1, immediate dephosphorylation of Atg13 and ULK, and facilitating the formation of pre-autophagosomal structures (PAS). Beclin-1, a key factor in cellular autophagy, is involved in the processes of autophagosome membrane formation. The kinase activity of ULK-1 is required for autophagy induction, and it mediates the pro-autophagy VPS34-Atg14-Beclin-1 complex (Class III phosphatidylinositol 3-kinase complex) formation via phosphorylating Beclin-1^33,34^. The generation of PI3P though the Class III PI3K complex and Atg9 mediates the accumulation of several other Atg proteins onto PAS for assembly whose action is to generate the isolation membrane, and it ultimately completes the formation of an autophagosome^34,35^. LC3 is involved in the autophagosome membrane extension phase. It primarily includes the Atg12–Atg5–Atg16 system and the phosphatidylethanolamine (PE)-microtubule associated protein 1 light chain 3 (MAPlLC3B, abbreviated as LC3) system. Atg12–Atg5–Atg16L is located in the outer membrane of autophagosomes and promotes the formation of Atg8-PE. The amount of Atg8-PE determines the size of the autophagosome and plays a critical role in the expansion of autophagosomes^36^. Additionally, Atg4 cleaves the LC3 precursor (Atg8) to form LC3-I that is then covalently bound to PE and converted to lipid-soluble LC3-II via catalysis by Atg7 and Atg3^37^. LC3-II, localized to the autophagosome membrane, is the first marker of mammalian autophagosome membranes^38^. Thus, Beclin1, the ratio of LC3-II/LC3-I, and the number of autophagosomes serve as indicators of autophagy activity. The ubiquitin-binding protein, P62, one of the important markers for selective autophagy, is a receptor protein that disassembles misfolded proteins^39^. The N-terminal PB1 structural domain of P62 forms a complex via the conjunction with LC3-II that promotes the generation of autophagic vesicles and the following degradation in the lysosome along with other protein aggregates^40,41^. Consequently, the activation of autophagy decreases the expression of the intracellular P62 protein (a schematic of autophagy is shown in Fig. 10). The observation indexes in our study included autophagic vesicles, p-mTOR/mTOR, P62, Beclin1, and LC3-II that can all objectively measure the occurrence and intensity of autophagy.

Autophagy and apoptosis are two essential cellular processes that enable cells to respond to cellular damage caused by various stressors^42^, and it is important to maintain a balance between these two processes to ensure cell survival and proper functioning. Excessive apoptosis can lead to cell death after stress during ischemia, hypoxia, and trauma. Autophagy is the first response to cellular damage and is dedicated to remedying various injuries^42^. Cellular stresses, such as nutrient deficiency, can trigger autophagy, allowing cells to regulate their internal components and adapt to their environment. Autophagy plays a dual role in cell survival or death, depending on the cellular environment^43^. Under stress conditions, autophagy is activated and is considered a pro-survival response^44^, however, excessive upregulation of autophagy can lead to cell death^45,46^.

Due to the complex relationship between autophagy and apoptosis. We subsequently investigated the mTOR-mediated autophagy pathway in the hippocampus of the ACMP rats. The results showed that the p-mTOR/mTOR ratio and the P62 protein levels were decreased in the hippocampi of rats in the CO group, while the expressions of Beclin1 and LC3-II were increased. In addition, a small number of autophagosomes and obviously swollen mitochondria were observed under electron microscopy. These data suggested that delayed brain injury after CO exposure was accompanied by autophagy, but this may not be enough to eliminate the damage to neurons caused by CO poisoning. In contrast, autophagy markers were further upregulated in the hippocampi of rats in the NaB group compared that of the CO group, and an increase in the number of autophagic vacuoles and a decrease in mitochondrial swellings were observed. These results suggested that sodium butyrate further promoted autophagy and was beneficial for improving CO-induced brain damage. It is worthy to note that the further activation of autophagy by sodium butyrate did not cause additional cellular damage. This suggested that sodium butyrate may attenuate hippocampal injury in ACMP rats via the upregulation of autophagy.

HDACs are an important class of epigenetic regulatory enzymes. They can remove the acetyl group from the lysine amino group of a target protein, thereby affecting gene transcription or altering protein activity. There are 11 known HDACs that can be categorized into classes, namely, class I (HDAC1, 2, 3, and 8), class IIa (HDAC4, 5, 7, and 9), class IIb (HDAC6 and 10), class III (SIRTs), and class IV HDAC (HDAC11)^47^. HDAC1, HDAC2, and HDAC3 are the most abundant HDACs in the hippocampus that activate the NF-κB signaling pathway and are involved in neuroinflammation^47,48^. In particular, HDAC1 plays a crucial role in promoting neuroinflammation^49,50^. Microglia are resident cells in the central nervous system (CNS) and are primarily involved in neuroinflammation. It has been found that LPS triggers the overexpression of HDACs in microglia, with HDAC1 being the most significantly elevated. HDACi decreases the release of inflammatory factors from microglia after LPS treatment^51^, In this study, we found that the HDAC1 protein levels were elevated at all-time points after CO poisoning. Sodium butyrate treatment attenuated brain injuries in the poisoned rats and decreased HDAC1 expression. CO poisoning-induced hypoxia resulted in elevated HDAC1 proteins, and HDAC1 overexpression may be involved in neuroinflammation aggravating CO-induced brain injury. Additionally, the inhibition of HDAC1 protein expression using sodium butyrate may also be a mechanism for its neuroprotective effect. However, the specific mechanism by which HDACs are involved in carbon monoxide poisoning-induced brain injury remains unclear and requires further study.

In conclusion, we found that the sodium butyrate treatment upregulated autophagy in the hippocampus of CO-exposed rats, inhibited neuronal apoptosis, and attenuated neurological injury. Therefore, sodium butyrate may be an effective therapeutic agent for the treatment of DEACMP.

## Materials and methods

### Experimental animals

Male adult Sprague–Dawley rats (aged 9–10 weeks, weighing 160–220 g, and specific-pathogen-free grade) were purchased from the Animal Center of North Sichuan Medical College (Quality Certificate No: SYXK (Chuan) 2018-076, License No: SYXK (Chuan) 2018–18). The rats were housed at the Department of Laboratory Animals in individual cages with independent ventilation systems and maintained under a constant standard condition: room temperature (21 ± 2 °C) and humidity (50 ± 15%), 12-h light/dark cycle, and free access to water and food. In our study, all of the experiments were approved by the welfare and ethics review committee of animal experiments of the North Sichuan Medical College [animal ethics review number: NSMC-2023047]. This study was conducted in compliance with the Animal Research: Reporting of In Vivo Experiments (ARRIVE) guidelines and conducted according to the Guidelines for the Animal Center of North Sichuan Medical College.

### Reagents

Carbon monoxide was purchased from the Chengdu Xindu Jinnengda Gas Co. (China). sodium butyrate was purchased from the Shanghai Macklin Biochemical Technology Co. (China). The BCA protein assay kit was purchased from Solarbio (China). The anti-HDAC1, anti-NeuN, anti-mTOR, anti-p-mTOR, anti-P62, anti-Beclin1, anti-LC3B, anti-Bax, anti-Bcl-2, anti-β-actin primary antibodies, and HRP-labeled goat anti-rabbit secondary antibodies were purchased from Affinity (USA). CoraLite594-conjugated Goat Anti-Rabbit IgG (1:250) was purchased from Proteintech (USA).

### Basic training and screening using the Morris water maze test

After 1 week of adaptive feeding, all of the rats in the present experiment were trained using the Morris water maze test. The Morris water maze consisted of a circular pool with a diameter of 2 m and a video system. The circular tub was divided equally into four quadrants. Black ink was added to the pool to create an opaque water. A target platform fixed 2 cm below the water surface was placed in the center of one of the quadrants. Rats were randomly placed into the water facing the sidewall in one of the quadrants. The trials of the rats were recorded immediately using a video system. If the rat was able to find the platform in 60 s, the time taken to climb the platform was recorded as the swimming latency. After the rat searched the platform, it was allowed to remain there for 15 s to strengthen its memory of the platform. In contrast, the escape latency was recorded as 60 s. The rats were placed into the four quadrants in sequence, and the average escape latency was calculated. On the sixth day, the data were used as the baseline result, and rats that were unable to climb the platform over 60 s were eliminated from the study.

### Establishment of the animal model

According to a previous protocol^52^, the CO inhalation method was used to establish the ACOP model. In brief, the rats in the CO and NaB groups were placed in a Plexiglas container (70 cm × 50 cm × 45 cm) with entrance and exit taps and exposed to 1000 ppm CO gas for 40 min, followed by the inhalation 3000 ppm for another 20 min to induce CO intoxication. A CO sensor was installed in the chamber to monitor the CO concentration. After the rats lost consciousness, they were moved out of the boxes and exposed to fresh air to recover consciousness. At the same time, the rats in the control group were exposed to normal air for 60 min in the aforementioned container.

### Experimental groups

In total, 72 rats that completed the basic water maze training successfully were assigned randomly to three groups: (1) the normal group (control): rats were exposed to air for 60 min; (2) the CO poisoning group (CO): rats were exposed to CO as described above; and (3) the sodium butyrate treatment group (NaB): rats were exposed to CO as described above and were intraperitoneal injected with 500 mg/kg of sodium butyrate. There were 24 rats in each group, and the 24 rats were separated into three subgroups randomly and equally based on time points of one, seven, and 14 days. Next, three rats in each subgroup were randomly selected for hematoxylin and eosin staining, Nissl staining, immunochemical staining,and TUNEL staining. The remaining five rats were used for transmission electron microscope and the western blot assay.

### Carboxyhemoglobin assessment

Immediately after CO exposure and before sodium butyrate administration, blood samples were obtained from the tail veins and heparinized. The modified, dual-wavelength COHb quantitation method was used to determine the blood concentrations of COHb to confirm the animal model of CO-poisoning^53^. First, 0.1 mL of blood samples was added to 20 mL of 0.4 M ammonium hydroxide and mixed evenly. This was followed by the addition of 20 mg of sodium dithionite and mixed evenly. Next, we measured the absorbances (A) at 535 and 578 nm using a spectrophotometer calibrated (Shimadzu) over 10 min. The blood levels of COHb were calculated using the following equation: COHb (%) = (2.44 × A535 nm/A578 nm − 2.68) × 100.

### Sodium butyrate intervention

Sodium butyrate was dissolved in ultrapure water to form a concentration of 200 mg/ml. After being removed from the Plexiglas chamber for 30 min, each of the rats in the NaB group was injected intraperitoneally with 500 mg/kg of sodium butyrate^54,55^, once a day and at the same time of day beginning on day one to day 14 after the animal model construction. An equivalent volume of 0.9% normal saline was administered to the rats in the CO group through daily intraperitoneal injections. The control group was handled in the same manner as the CO group.

### Morris water maze test

On days one, seven, and 14 after the animal model construction, the Morris water maze (MWM) experiment was performed again using the same methods as the description of the basic training.

### Tissue sample collection

After the rats underwent the second Morris water maze experiment, 0.1% pentobarbital was administered via intraperitoneal injection for a deep anesthesia. The rats were then perfused with 0.01 M phosphate buffer solution (PBS) and 4% paraformaldehyde (PFA) transcardially. The skulls were opened, the entirety of the brains was removed. They were then postfixed in 4% PFA over 6 h. The brains were then dehydrated in gradient ethanol, transparented in dimethyl benzene, and finally embedded in paraffin. To test the expression of the target genes related to autophagy, the hippocampus tissues were placed in a tube and transferred to a freezer for storage at − 80 °C. To investigate the autophagosomes, the hippocampi tissues were immersed in 4% glutaraldehyde for storage at 4 °C.

### Western blot assay

The total protein was extracted from the hippocampal tissues. A total of 20 mg the sample was added to the protein lysate that consisted of 200ul of RIPA (Solarbio), 2 ul of phenyl methyl sulfonyl fluoride (PMSF,100 mM), and 2 ul of protein phosphatase inhibitors(All-in-one, 100×). After being crushed by an ultrasonic crusher, the tissues were lysed on ice for 60 min and then centrifuged at 12,000 r/min for 15 min at 4 °C. The supernatants were collected, and the protein concentration was determined utilizing the BCA protein assay kit. The samples were then mixed with the loading buffer, RIPA, and boiled for 10 min. Next, equal amounts of each sample were separated via SDS–polyacrylamide gel electrophoresis (PAGE), followed by being transferred to polyvinylidene fluoride (PVDF) membranes (Merck Millipore). Tailored PVDF membranes should be designed according to the target molecules. After blocking in Tris-buffered saline and Tween 20 solution (TBST) containing 5% skimmed milk powder for an hour at room temperature, this was incubated using a primary antibody (HDAC1 dilution 1:1000 mTOR dilution 1:1000; p-mTOR dilution1:1000; p62 dilution 1:1000; Beclin1 dilution 1:1000; LC3B dilution 1:1000; BAX dilution 1:1000; bcl-2 dilution 1:1000; and β-actin dilution 1:1000) at 4 °C overnight. On the second day, the membrane was incubated using HRP-labeled goat anti-rabbit secondary antibodies (Goat anti-rabbit, 1:5000,) at room temperature for 1 h. The membrane was then washed with TBST to remove any unbound antibody. Finally, ECL solution (Affinity, USA) was added to the membrane, and the target proteins were analyzed using the Bio-Rad ChemiDoc XRS + and image J software. The protein levels are presented as the ratio of the targeted proteins/β-actin.

### Hematoxylin and eosin staining

The histopathological evaluations were performed following the hematoxylin and eosin (H&E) staining. For this purpose, after taking out the entire brains, they were fixed in 4% PFA over 6 h. The samples were then sent to the pathology department of the Affiliated Hospital of North Sichuan Medical College, Nanchong, China. Pathological changes in the hippocampi were observed using an optical microscope (Olympus) under 200× magnification, and images were collected to evaluate the morphologic variations of the neurons in the hippocampi.

### Nissl staining (toluidine blue staining)

The paraffin sections were dewaxed with xylene and soaked in gradient ethanol of decreasing concentration for rehydration and washed with DDH2O, stained with toluidine blue (Solarbio) at 50 °C for 30 min, followed by a differentiation in 95% alcohol (brain tissues were differentiated until the Nissl bodies appeared deep blue and the background became pale blue or color-less). Next, the sections were dehydrated using 100% ethanol, transparented with xylene, and then air-dried and mounted with neutral resin. The CA1 areas of the hippocampi were observed under a light microscope at 200× magnification. A quantitative analysis of surviving neurons was performed using Image J software.

### Immunochemical and TUNEL staining

The tissue preparation for the immunochemical and TUNEL staining was the same as that for the Nissl staining. Next, tissue sections were submerged in a sodium citrate buffer and microwaved on high for 15 min, cooled naturally, and then endogenous peroxidase blockers were added for 20 min. After being washed with PBS, and the specimens were incubated with 5% BSA for an hour at room temperature. The primary antibodies were then applied to the sections at 4 °C overnight. The primary antibodies were rabbit anti-Neun (1:100, Affinity), rabbit anti-Beclin1 (1:100, Affinity), and rabbit anti-LC3B (1:100, Affinity). On the second day, the samples used for immunofluorescence were incubated with fluorophore-conjugated secondary antibodies (goat anti-rabbit antibody conjugated to Alexa 594, 1:250) for an hour at room temperature, and the slides were mounted with antibody fluorescent attenuation containing DAPI mounting medium. The tissue sections were then observed under a fluorescence microscope. The samples used for the immunohistochemical analysis were incubated at room temperature for 20 min with biotin-labelled goat anti- rabbit IgG (maxim) and streptomyces anti-biotin protein-peroxidase (maxim). The sections were then stained using 3,3′-diami-nobenzidine (maxim) and hematoxylin. The sections were observed under an optical microscope after dehydration, clearing, and mounting with neutral resin.

The TdT-mediated dUTP nick-end labeling (TUNEL) assay was performed to evaluate the apoptotic cells in the CA1 region (keygen) (n = 3/group). In brief, after washing in PBS, sections were submerged in a sodium citrate buffer and microwaved on high for 8 min, cooled naturally, and then proteinase K was added. The specimens were then incubated for 30 min at 37 °C. After washing with PBS, the TdT enzyme reaction solution and streptavidin-HRP epitope solution was applied to the sections for 30 min at 37 °C separately in a humified atmosphere devoid of light. After incubation, the samples were washed with PBS. The sections were then stained using 3,3′-diami-nobenzidine and hematoxylin. After dehydration, clearing, and mounting with neutral resin, the sections were observed under an optical microscope.

### Transmission electron microscope (TEM)

The hippocampi tissues were separated from the entire brains carefully and rapidly in a matrix surrounded by cold ice and sliced into 1 mm^3^ pieces. They were then immersed in 4% glutaraldehyde over 3 h at 4 °C. After being postfixed using 1% osmium acid for 90 min at 35 °C and dehydrated with gradient ethanol, the tissues were embedded in epoxy resin 812 and cut into ultrathin sections of 80 nm using an ultramicrotome (Leica UC7). The slices were then immersed in 3% acetic acid uranium and dyed for 15 min. This was followed by 6% lead citrate solution for 5 min. Finally, the ultrastructure was observed under a TEM (HITACHI HT7800).

### Statistical analysis

SPSS 26.0 was utilized for the data analysis, while Image J was employed for the image analysis. Statistical charts were drawn using GraphPad Prism 9.0. The mean ± standard deviation (± s) was utilized to express all of the measurement data. We utilized t-tests or analysis of variance (ANOVA) to compare the measurement data groups that adhered to normal distributions and nonparametric rank-sum tests for those that did not. Statistical significance was set at *p* < 0.05.

### Supplementary Information

Supplementary Figure S1.

Supplementary Figure S2.

Supplementary Figure S3.

Supplementary Figure S4.

Supplementary Figure S5.

Supplementary Figure S6.

Supplementary Figure S7.

Supplementary Figure S8.

Supplementary Figure S9.

Supplementary Figure S10a.

Supplementary Figure S10b.

Supplementary Figure S10c.

Supplementary Figure S11a–b.

Supplementary Figure S11c.

Supplementary Figure S12a–b.

Supplementary Figure S12c.

Supplementary Figure S13.

Supplementary Figure S14a.

Supplementary Figure S14b.

Supplementary Figure S14c.

Supplementary Figure S15a.

Supplementary Figure S15b.

Supplementary Figure S15c.

Supplementary Figure S16a–b.

Supplementary Figure S16c.

Supplementary Figure S17a–b.

Supplementary Figure S17c.

Supplementary Figure S18a–b.

Supplementary Figure S18c.

Supplementary Figure S19a–b.

Supplementary Figure S19c.

Supplementary Figure S20.

## Data Availability

All data generated or analyzed during this study are included in this published article and supplementary information files.
