# Supplementary figures and images for: Sodium butyrate exerts a neuroprotective effect in rats with acute carbon monoxide poisoning by activating autophagy through the mTOR signaling pathway
Source: Sci Rep. 2024 Feb 26;14:4610. doi: 10.1038/s41598-024-55198-z (PMC10897214; doi:10.1038/s41598-024-55198-z)

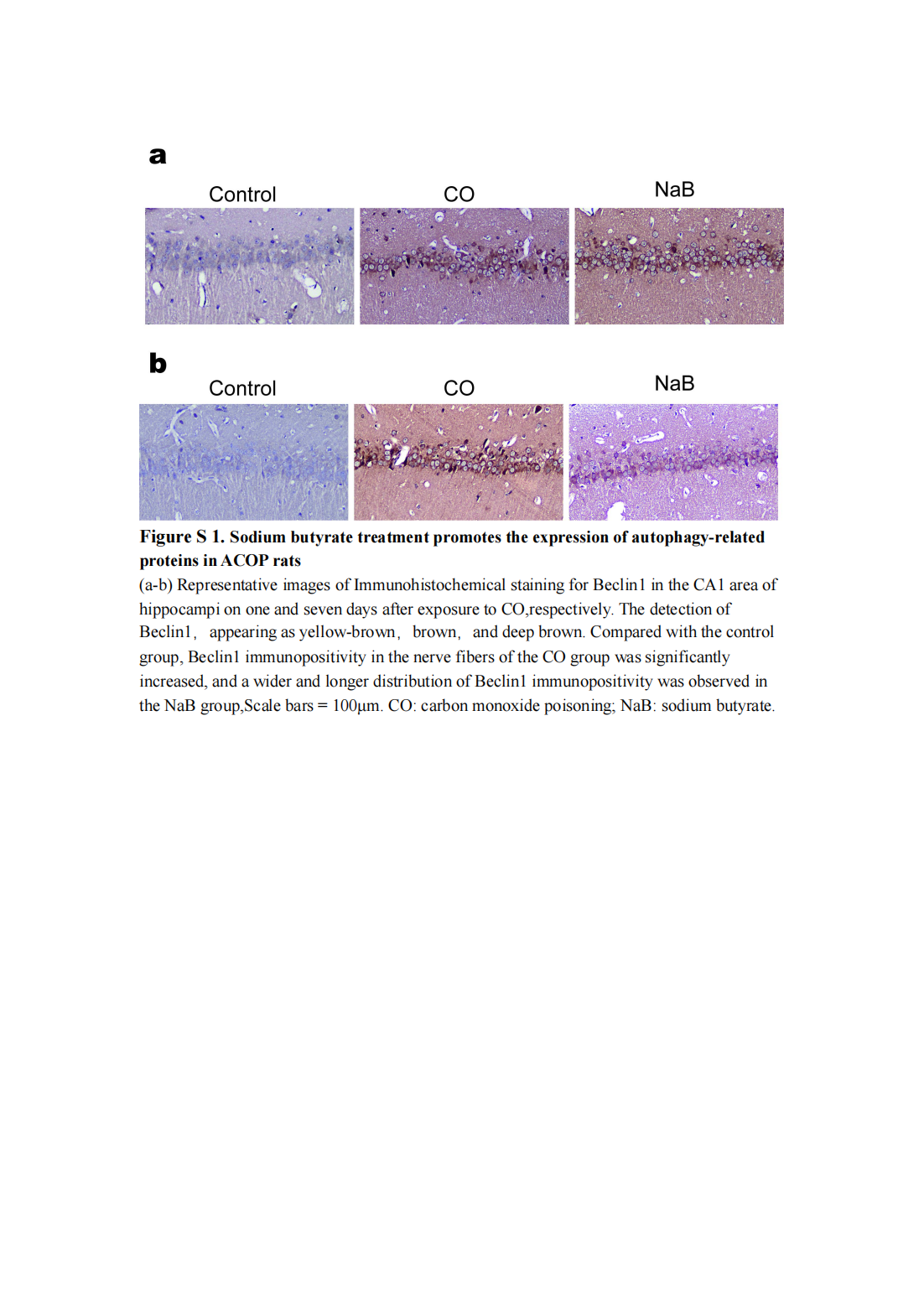

Supplement: Supplementary file 1 — Supplementary Figure S1. [file 41598_2024_55198_MOESM1_ESM.tif]

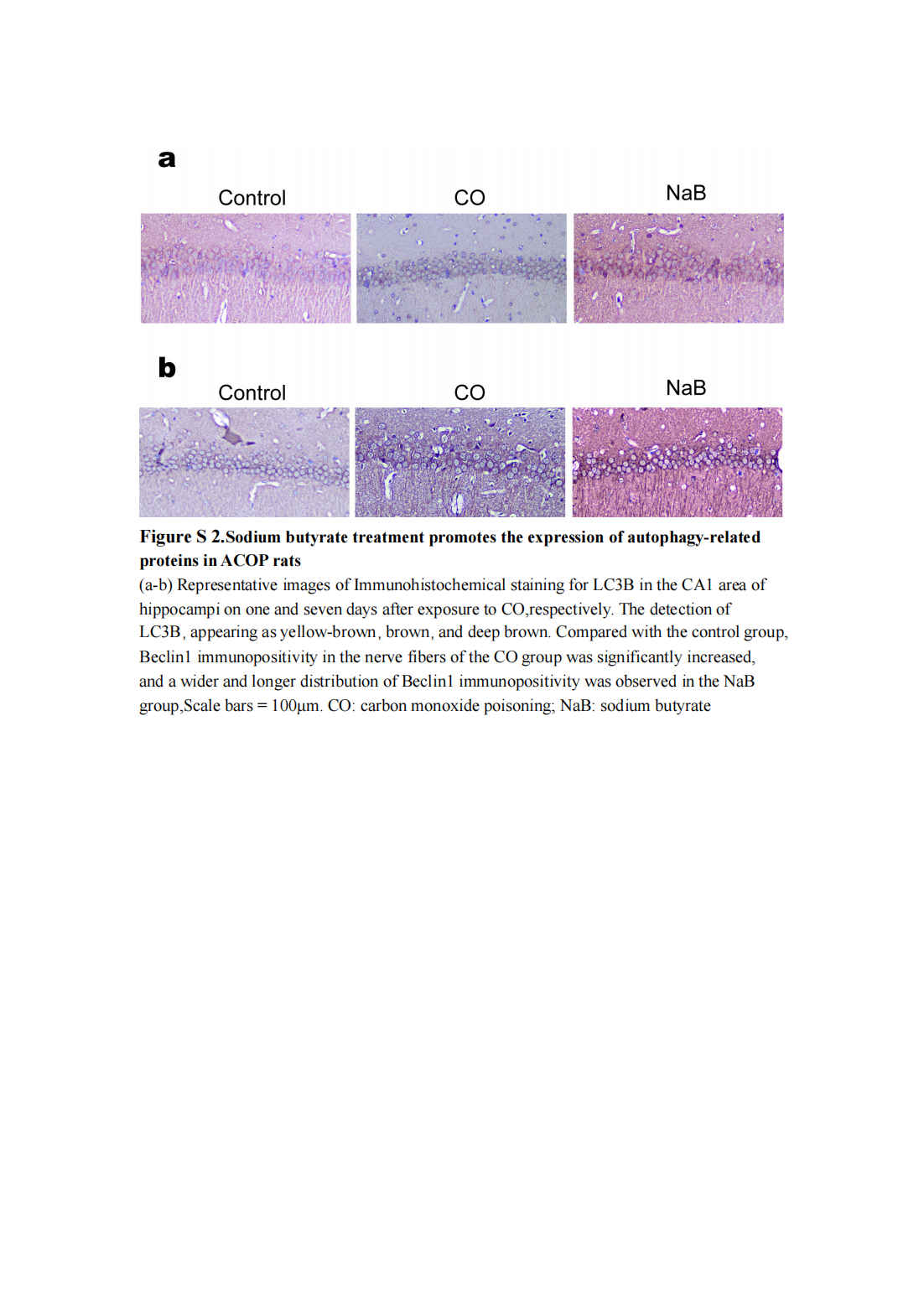

Supplement: Supplementary file 2 — Supplementary Figure S2. [file 41598_2024_55198_MOESM2_ESM.tif]

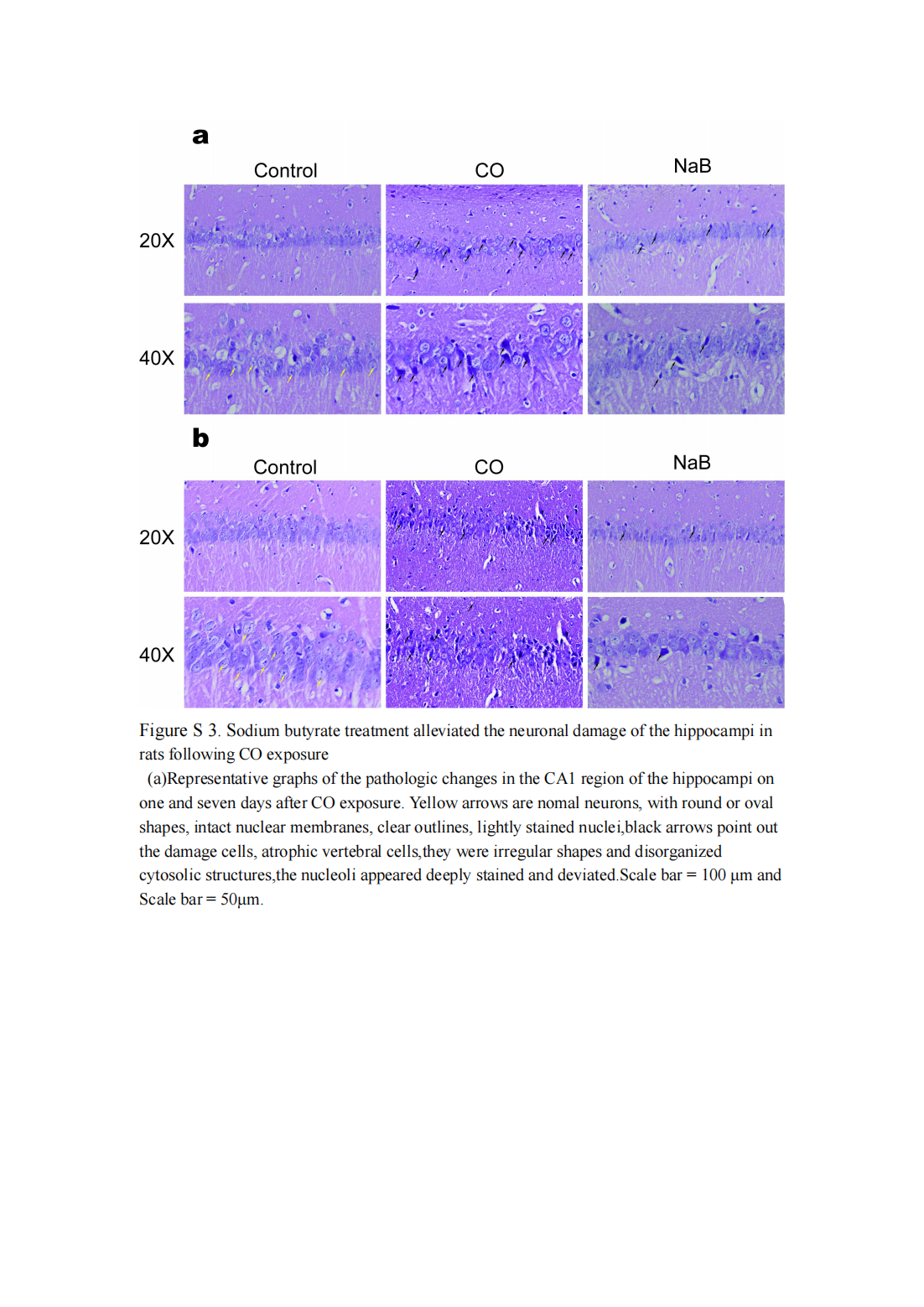

Supplement: Supplementary file 3 — Supplementary Figure S3. [file 41598_2024_55198_MOESM3_ESM.tif]

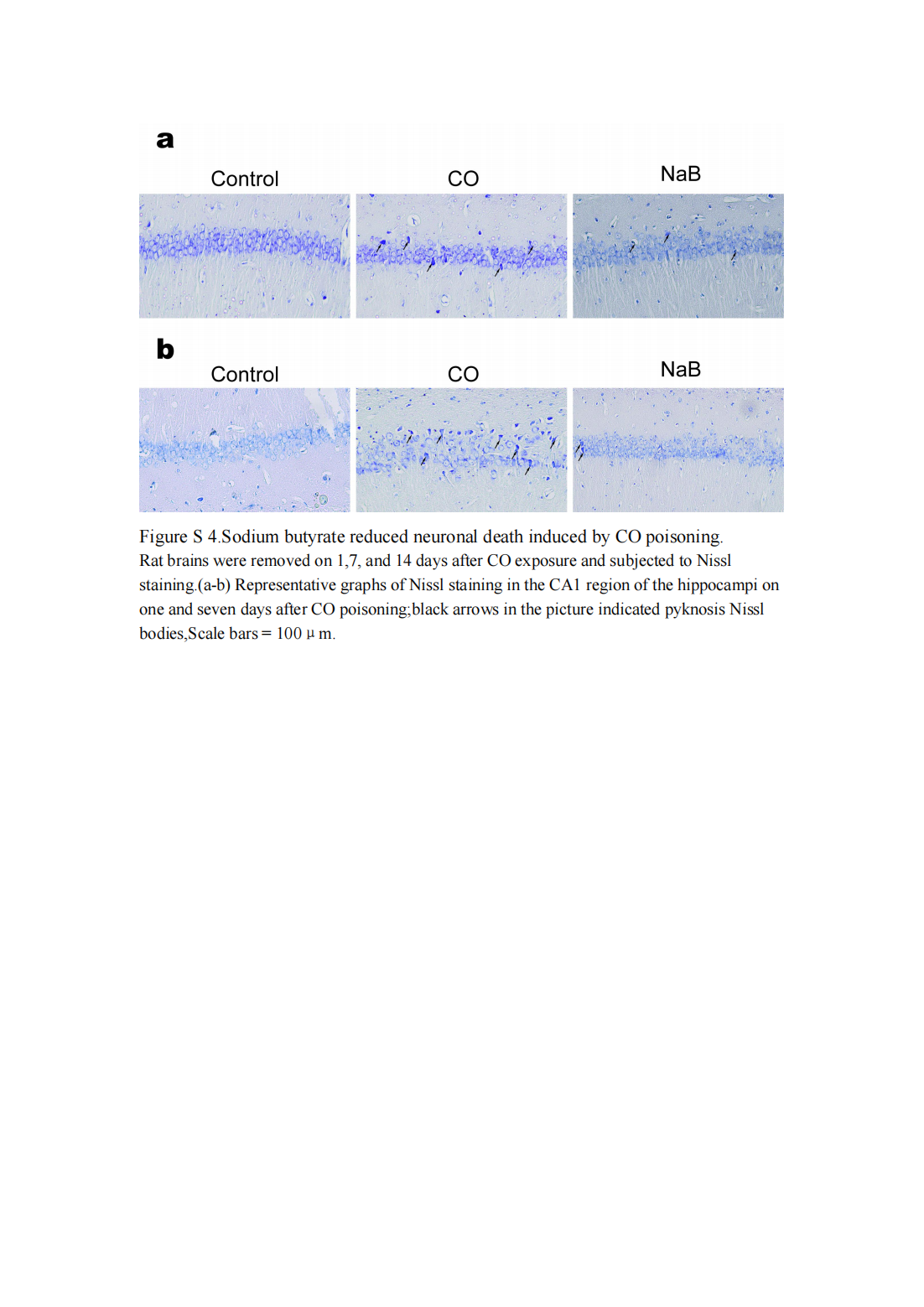

Supplement: Supplementary file 4 — Supplementary Figure S4. [file 41598_2024_55198_MOESM4_ESM.tif]

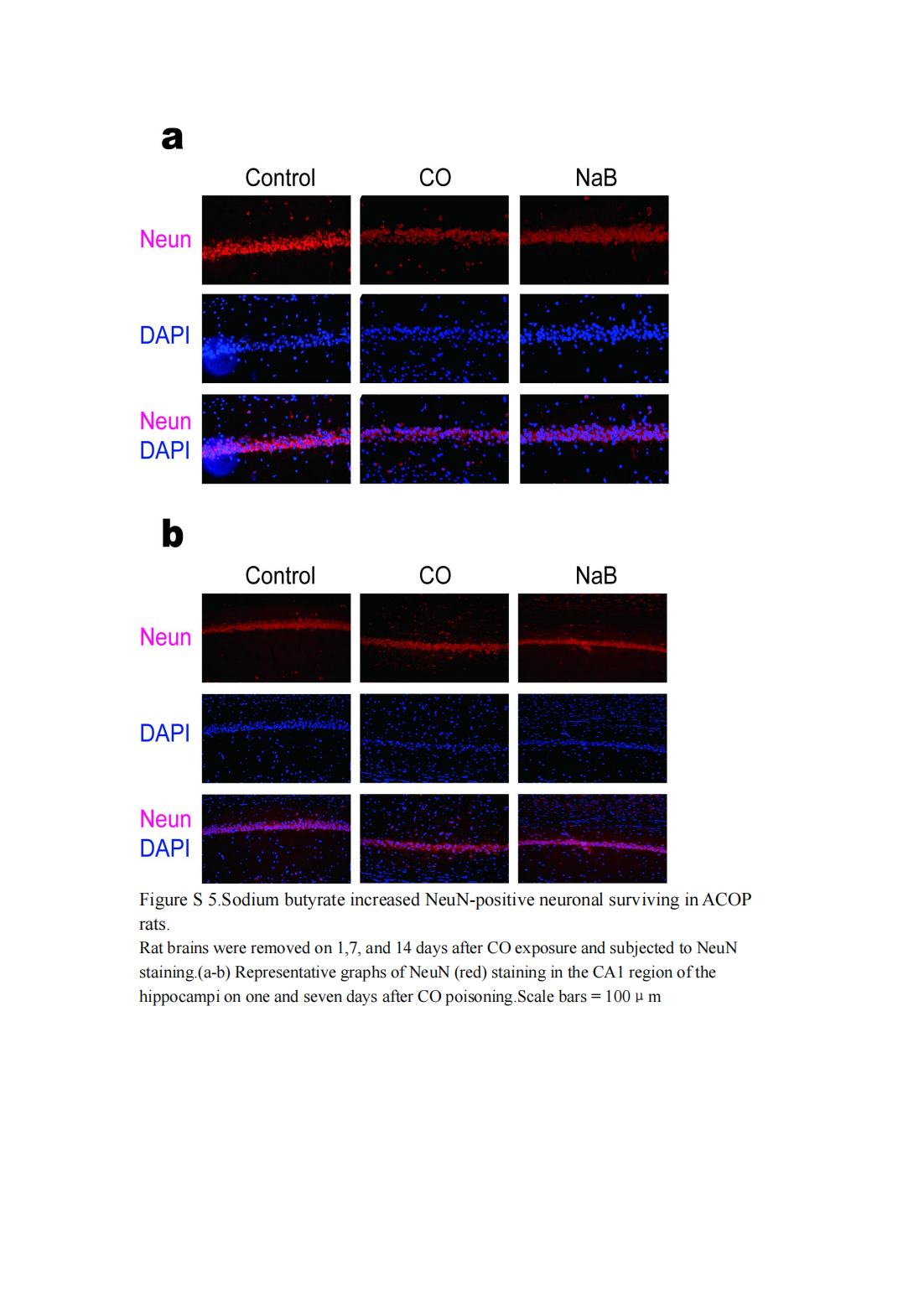

Supplement: Supplementary file 5 — Supplementary Figure S5. [file 41598_2024_55198_MOESM5_ESM.tif]

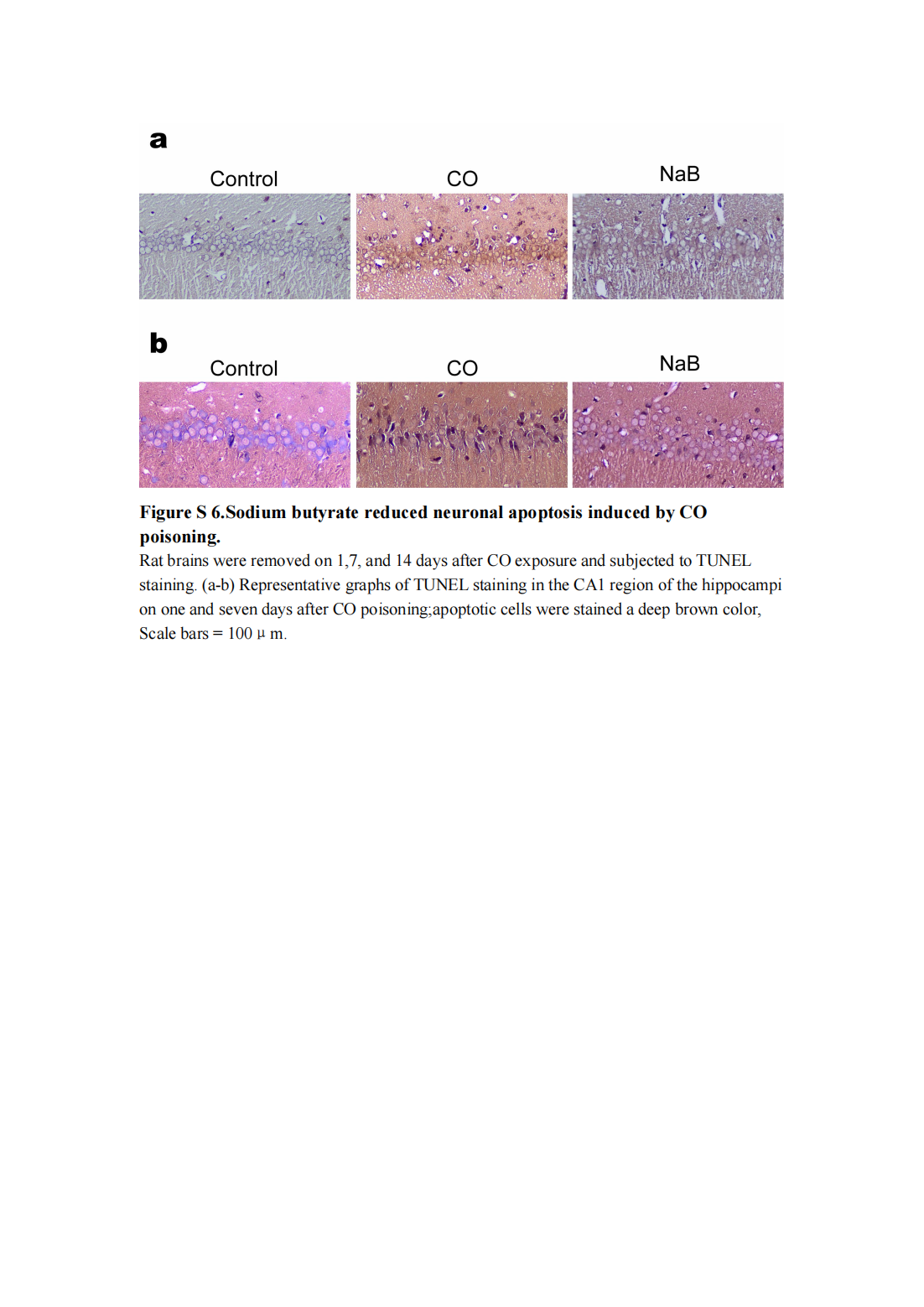

Supplement: Supplementary file 6 — Supplementary Figure S6. [file 41598_2024_55198_MOESM6_ESM.tif]

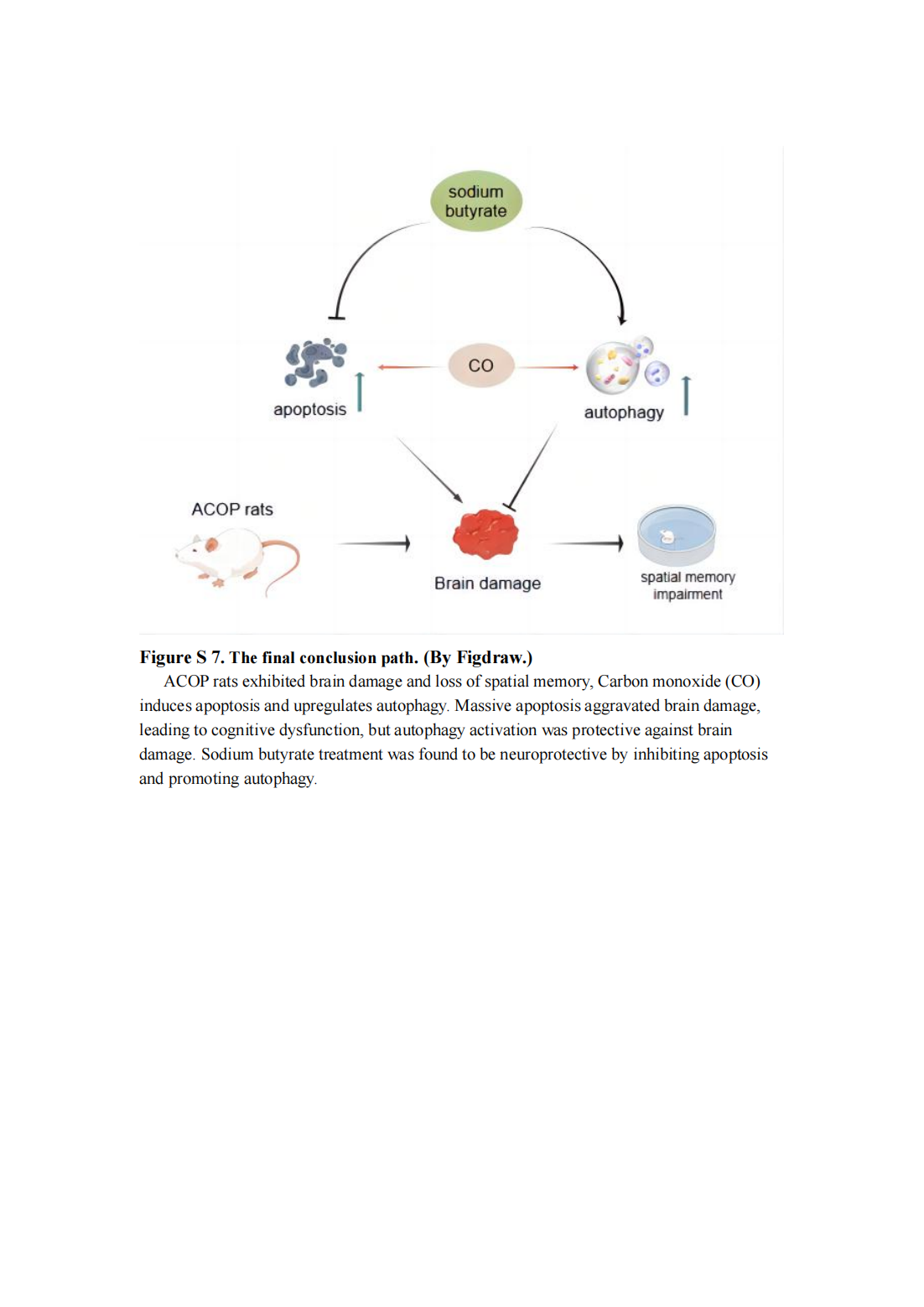

Supplement: Supplementary file 7 — Supplementary Figure S7. [file 41598_2024_55198_MOESM7_ESM.tif]

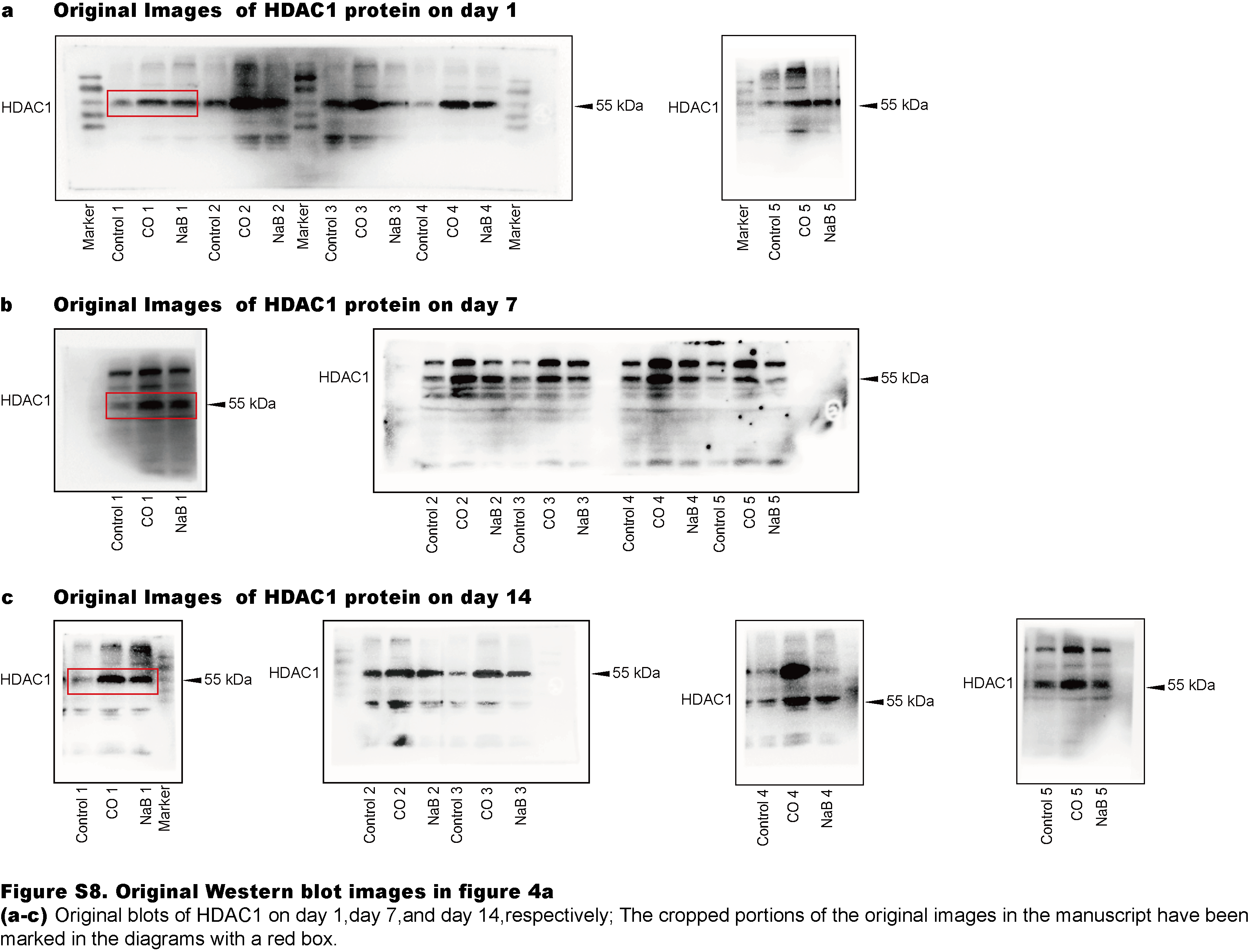

Supplement: Supplementary file 8 — Supplementary Figure S8. [file 41598_2024_55198_MOESM8_ESM.tif]

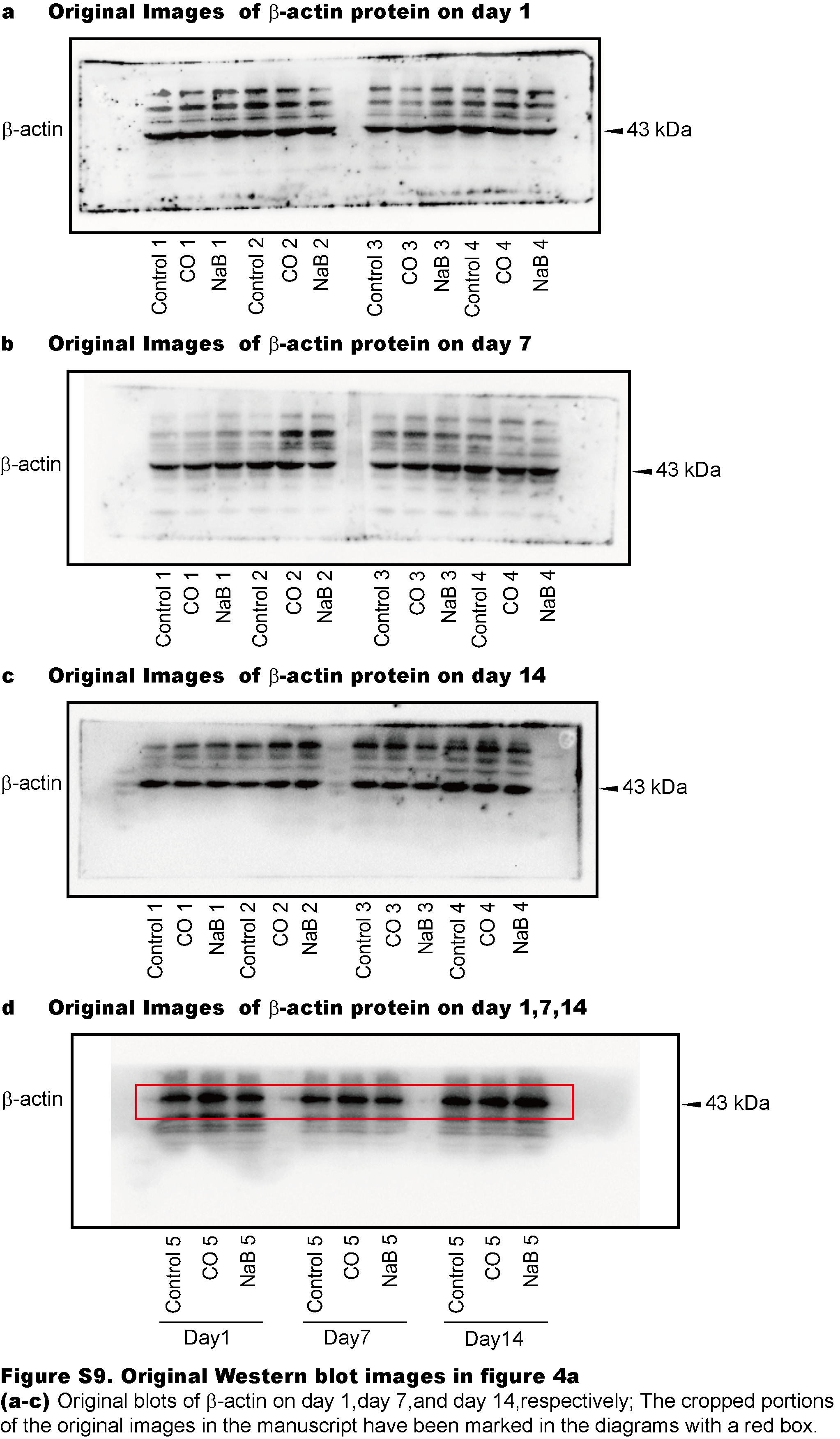

Supplement: Supplementary file 9 — Supplementary Figure S9. [file 41598_2024_55198_MOESM9_ESM.tif]

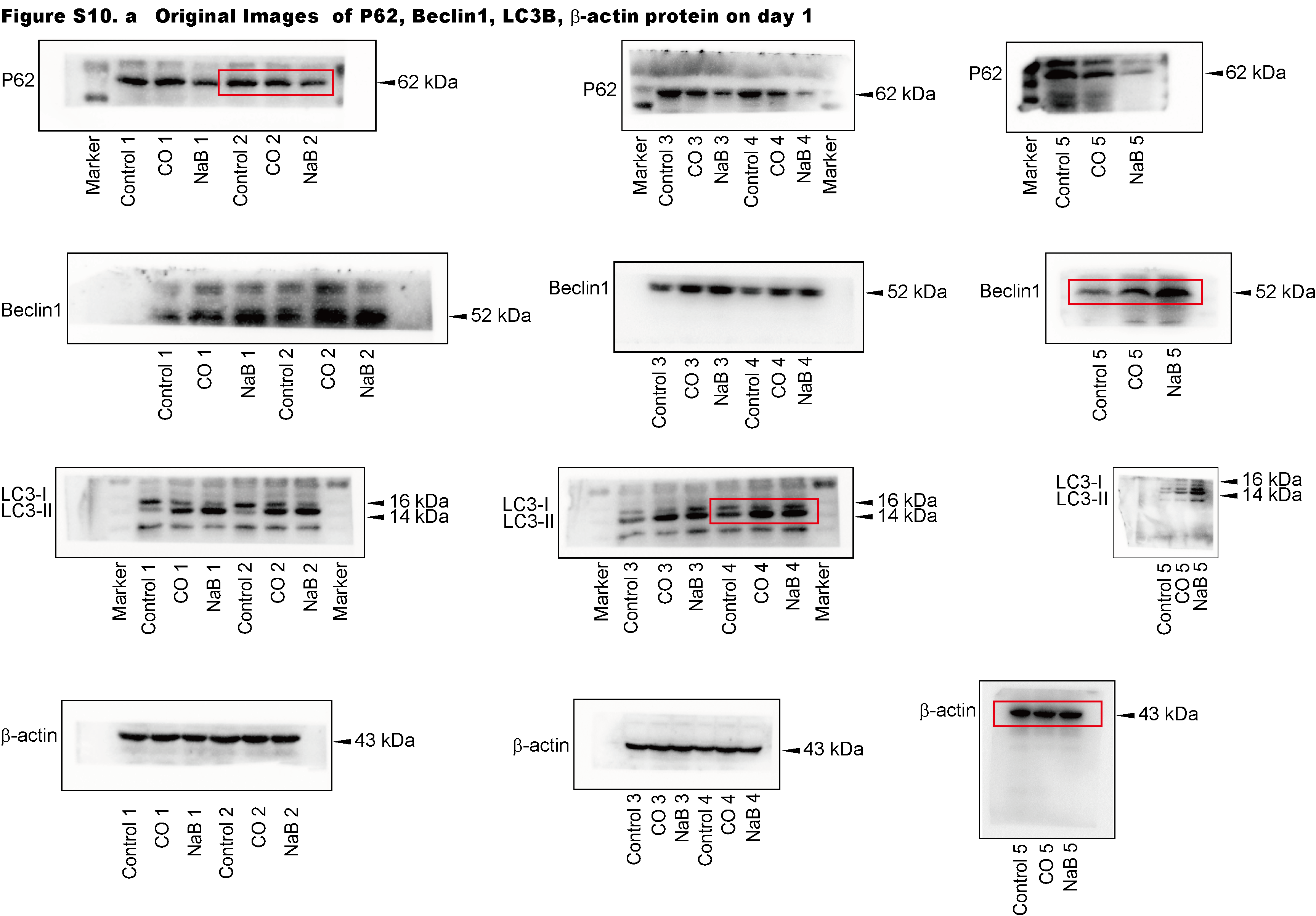

Supplement: Supplementary file 10 — Supplementary Figure S10a. [file 41598_2024_55198_MOESM10_ESM.tif]

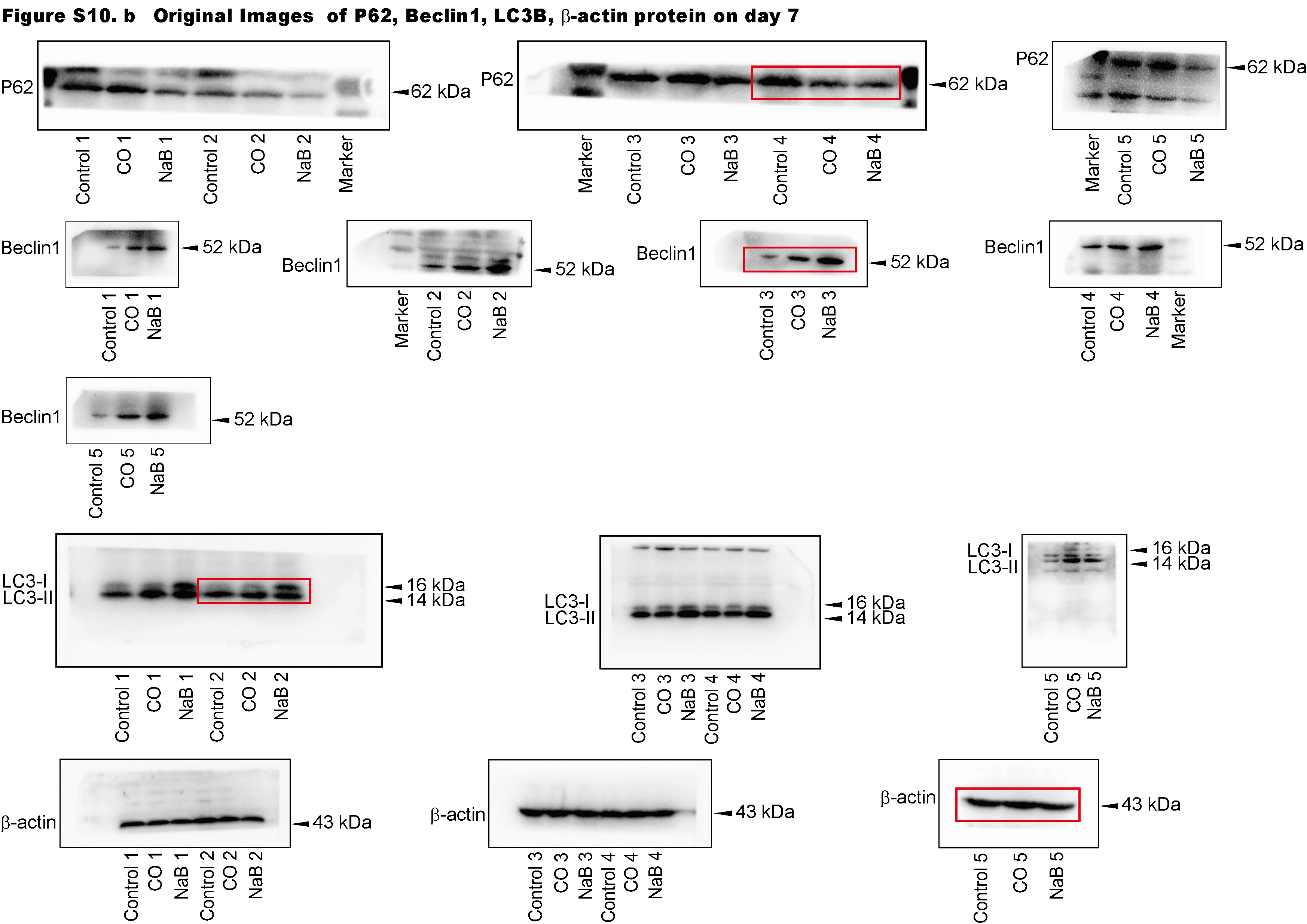

Supplement: Supplementary file 11 — Supplementary Figure S10b. [file 41598_2024_55198_MOESM11_ESM.tif]

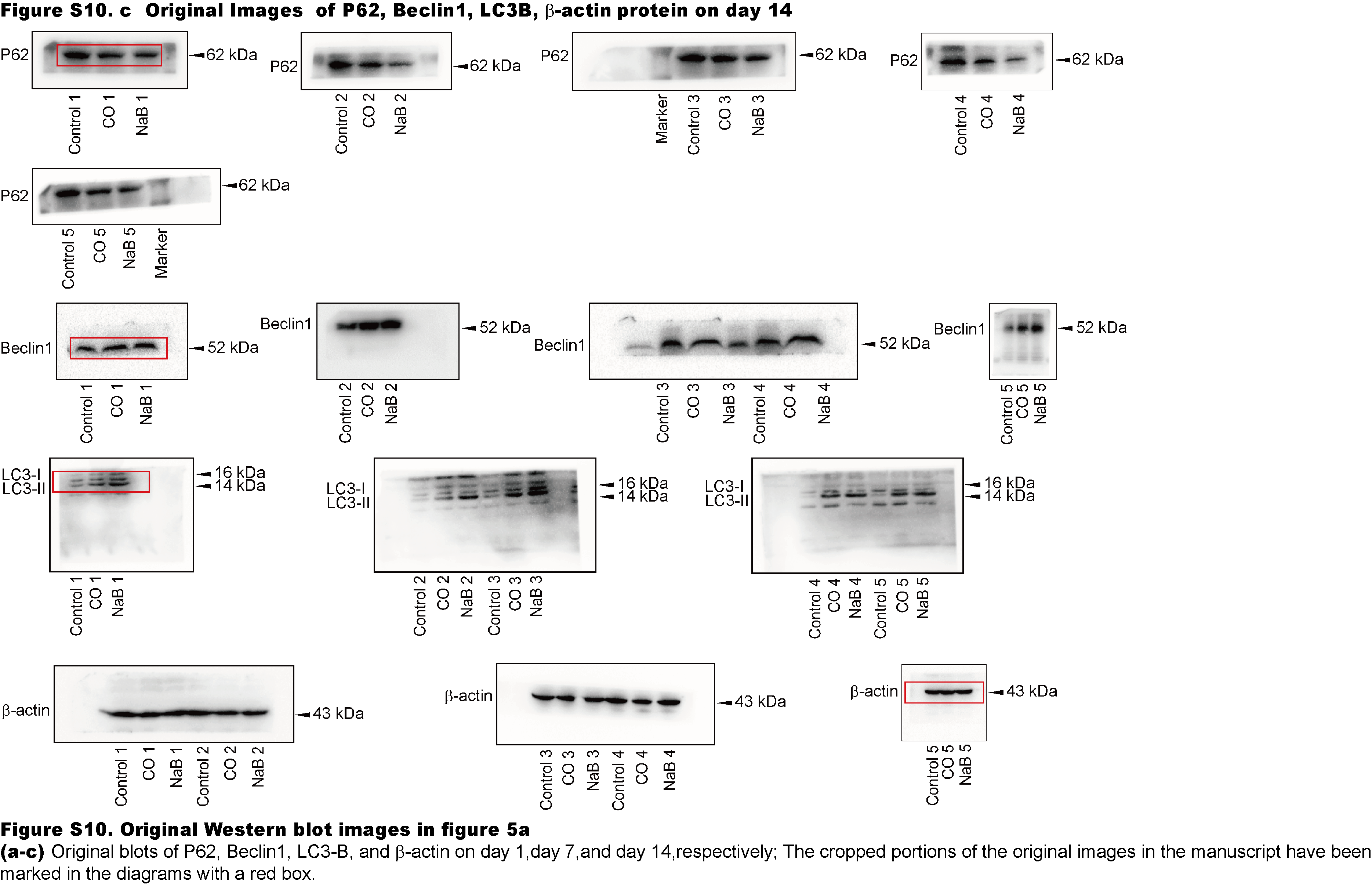

Supplement: Supplementary file 12 — Supplementary Figure S10c. [file 41598_2024_55198_MOESM12_ESM.tif]

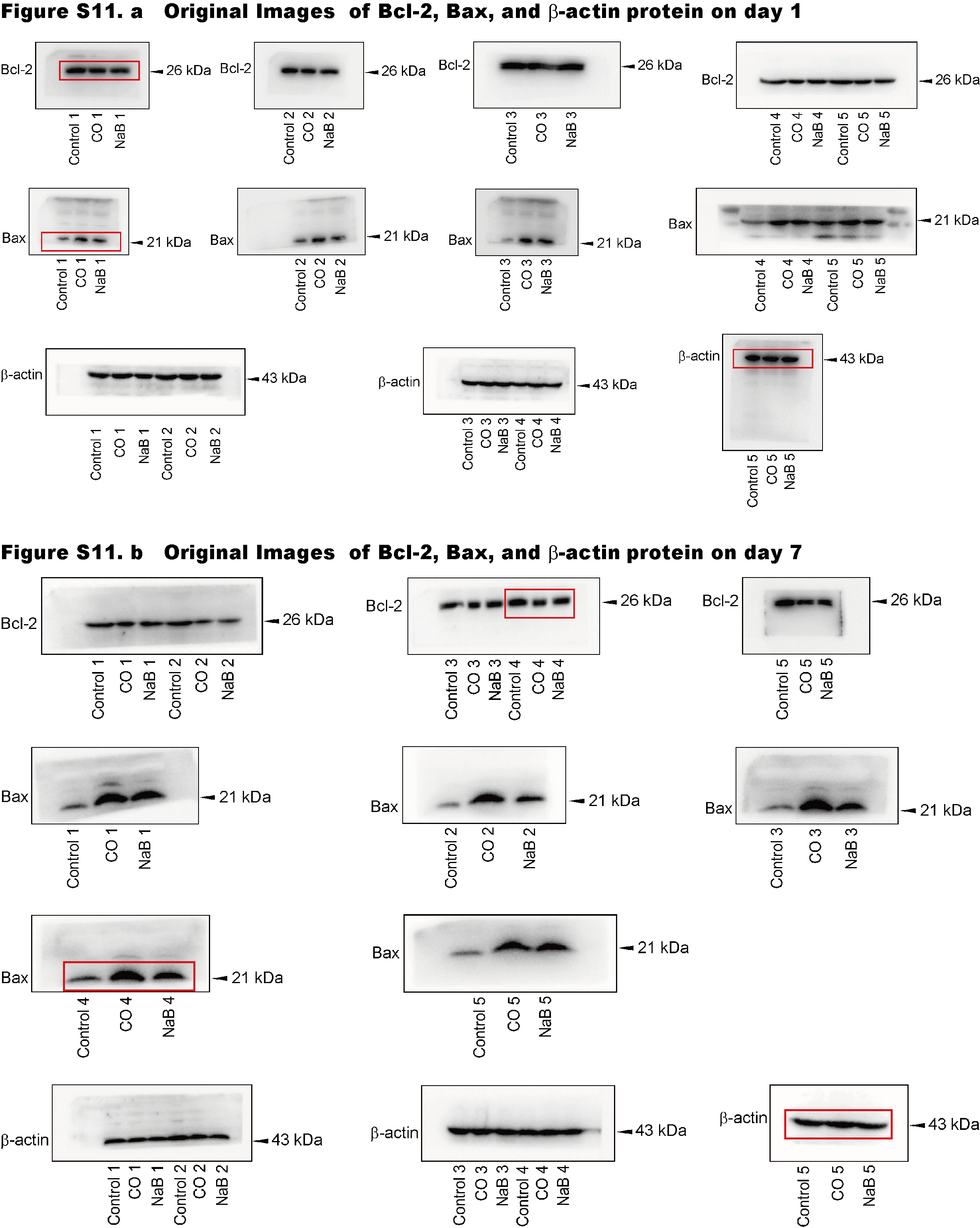

Supplement: Supplementary file 13 — Supplementary Figure S11a–b. [file 41598_2024_55198_MOESM13_ESM.tif]

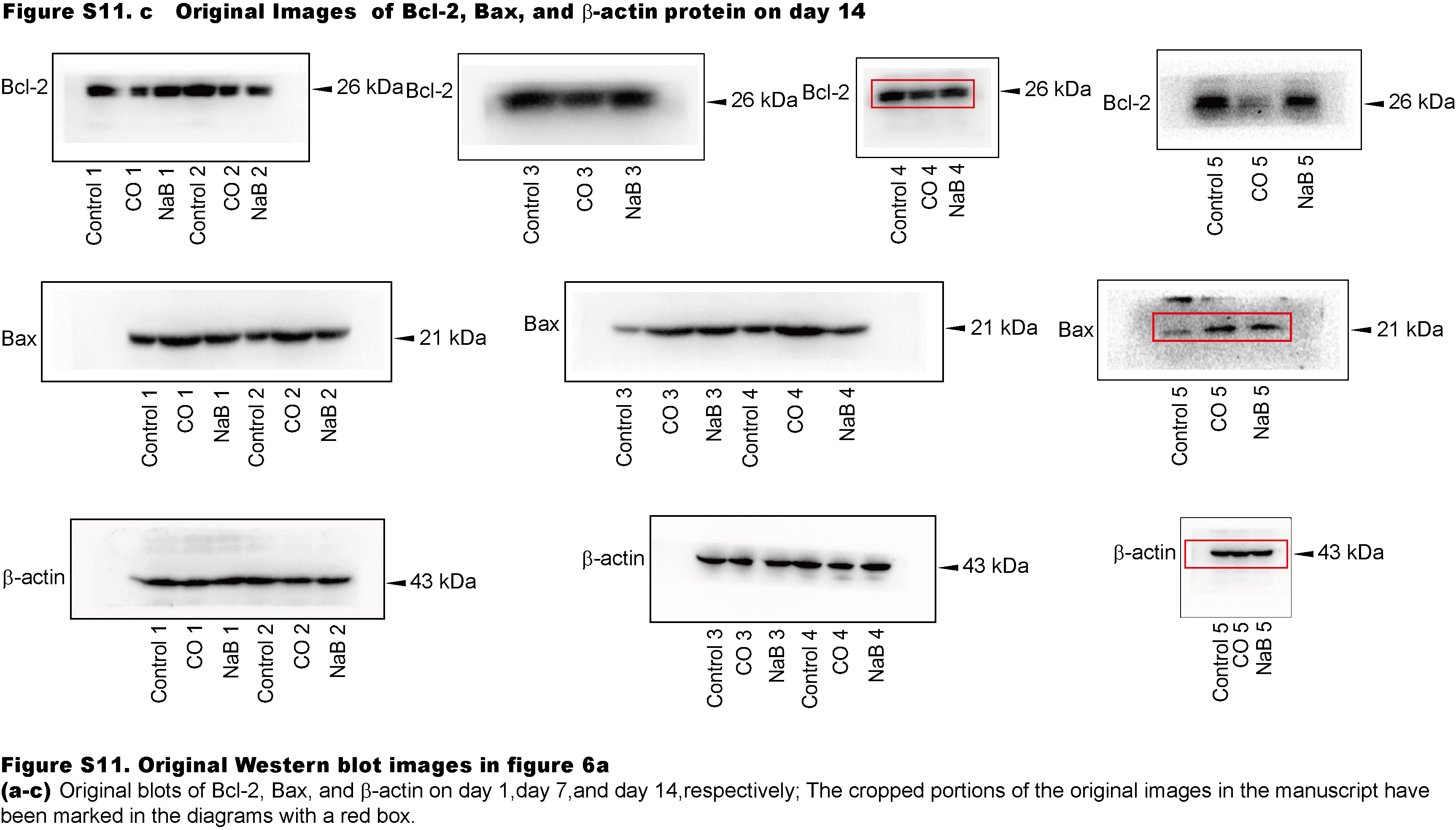

Supplement: Supplementary file 14 — Supplementary Figure S11c. [file 41598_2024_55198_MOESM14_ESM.tif]

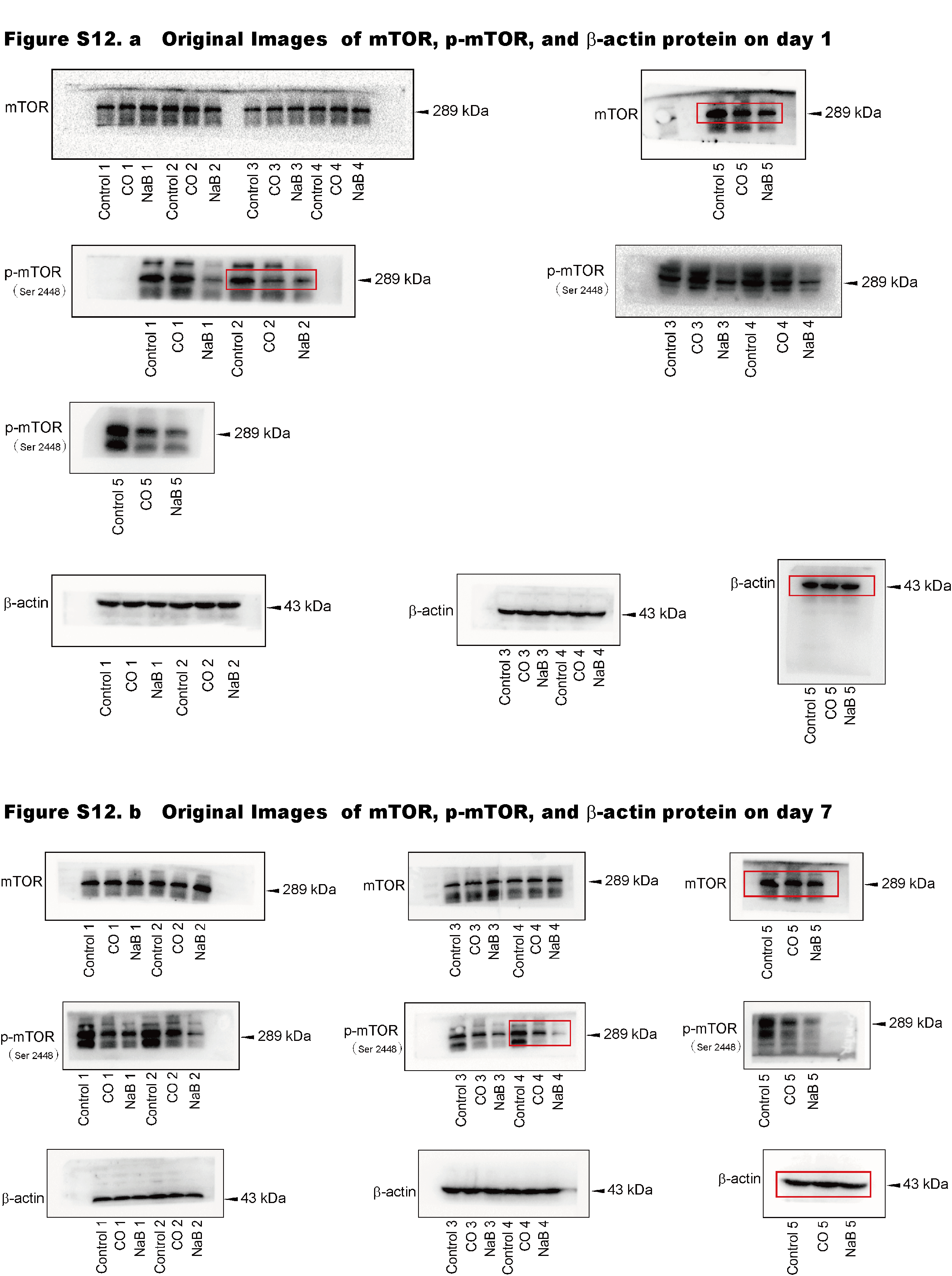

Supplement: Supplementary file 15 — Supplementary Figure S12a–b. [file 41598_2024_55198_MOESM15_ESM.tif]

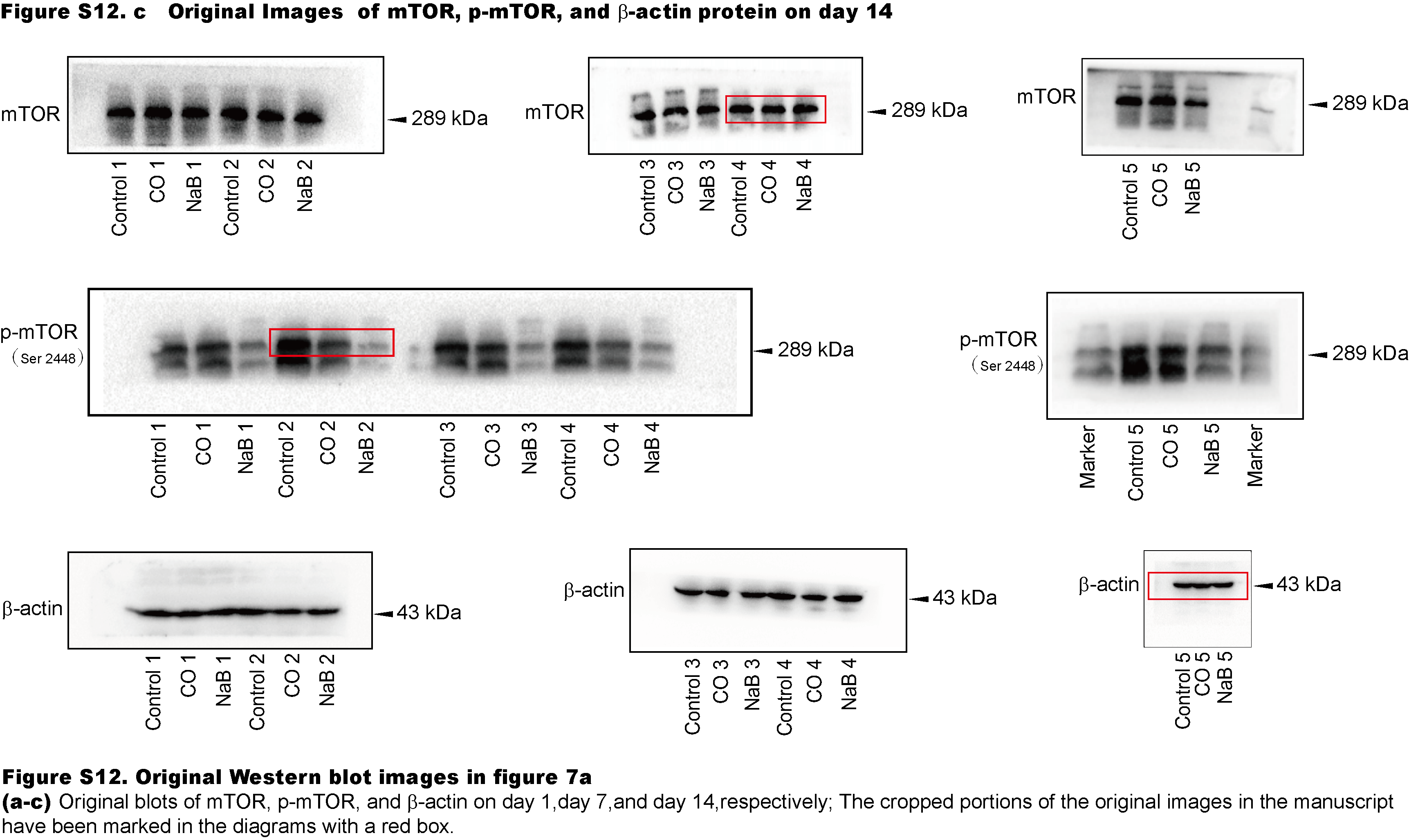

Supplement: Supplementary file 16 — Supplementary Figure S12c. [file 41598_2024_55198_MOESM16_ESM.tif]

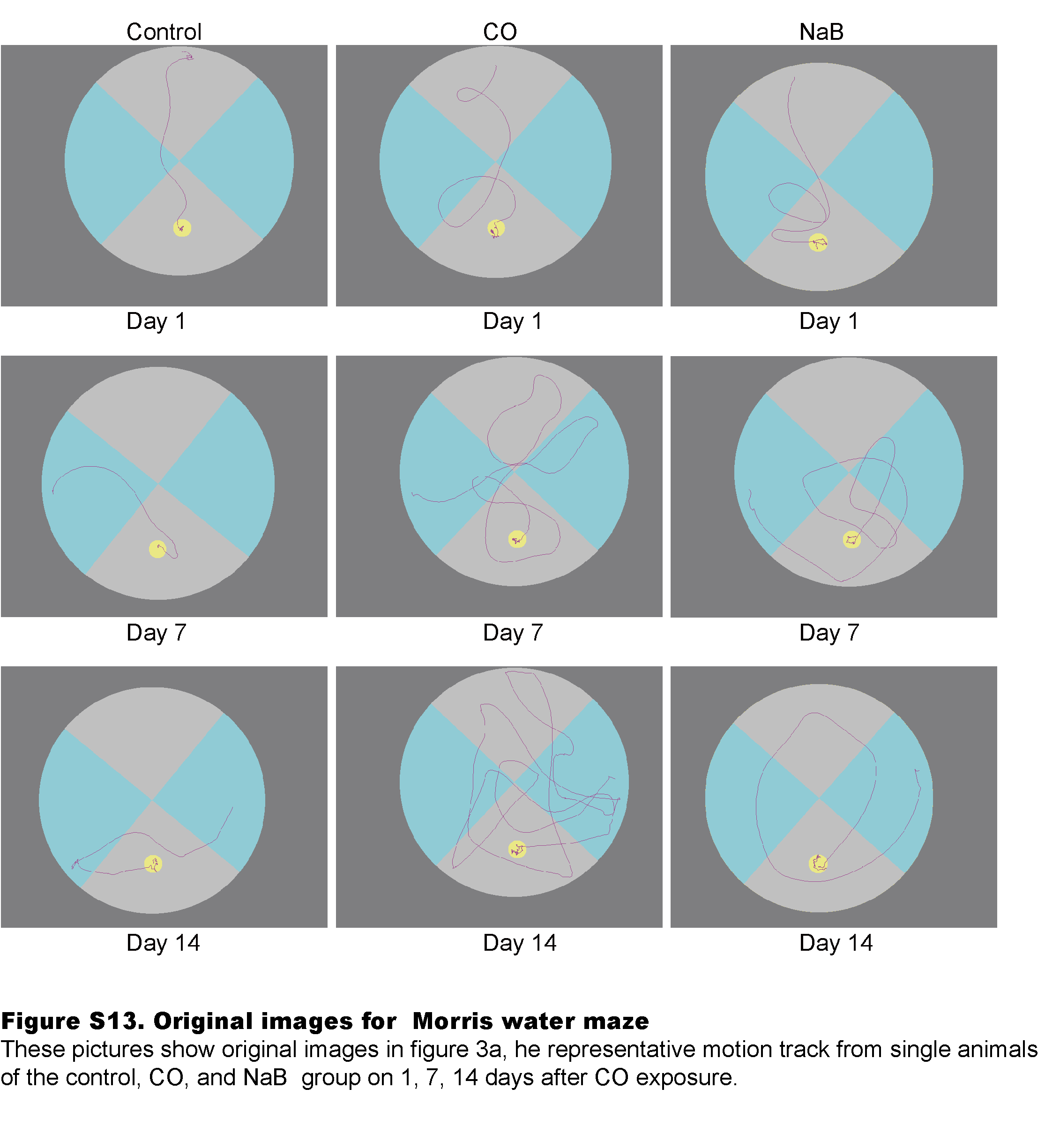

Supplement: Supplementary file 17 — Supplementary Figure S13. [file 41598_2024_55198_MOESM17_ESM.tif]

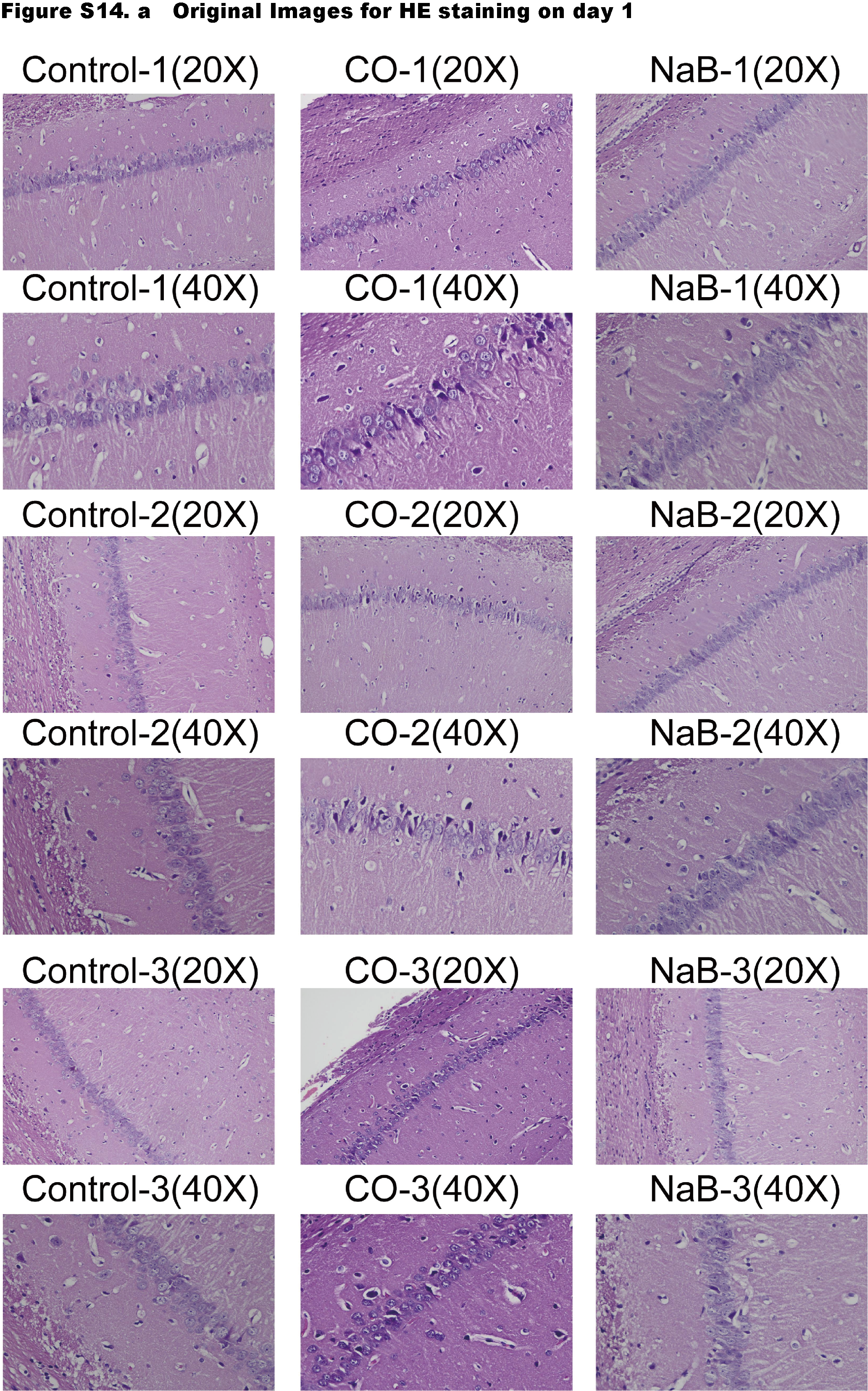

Supplement: Supplementary file 18 — Supplementary Figure S14a. [file 41598_2024_55198_MOESM18_ESM.tif]

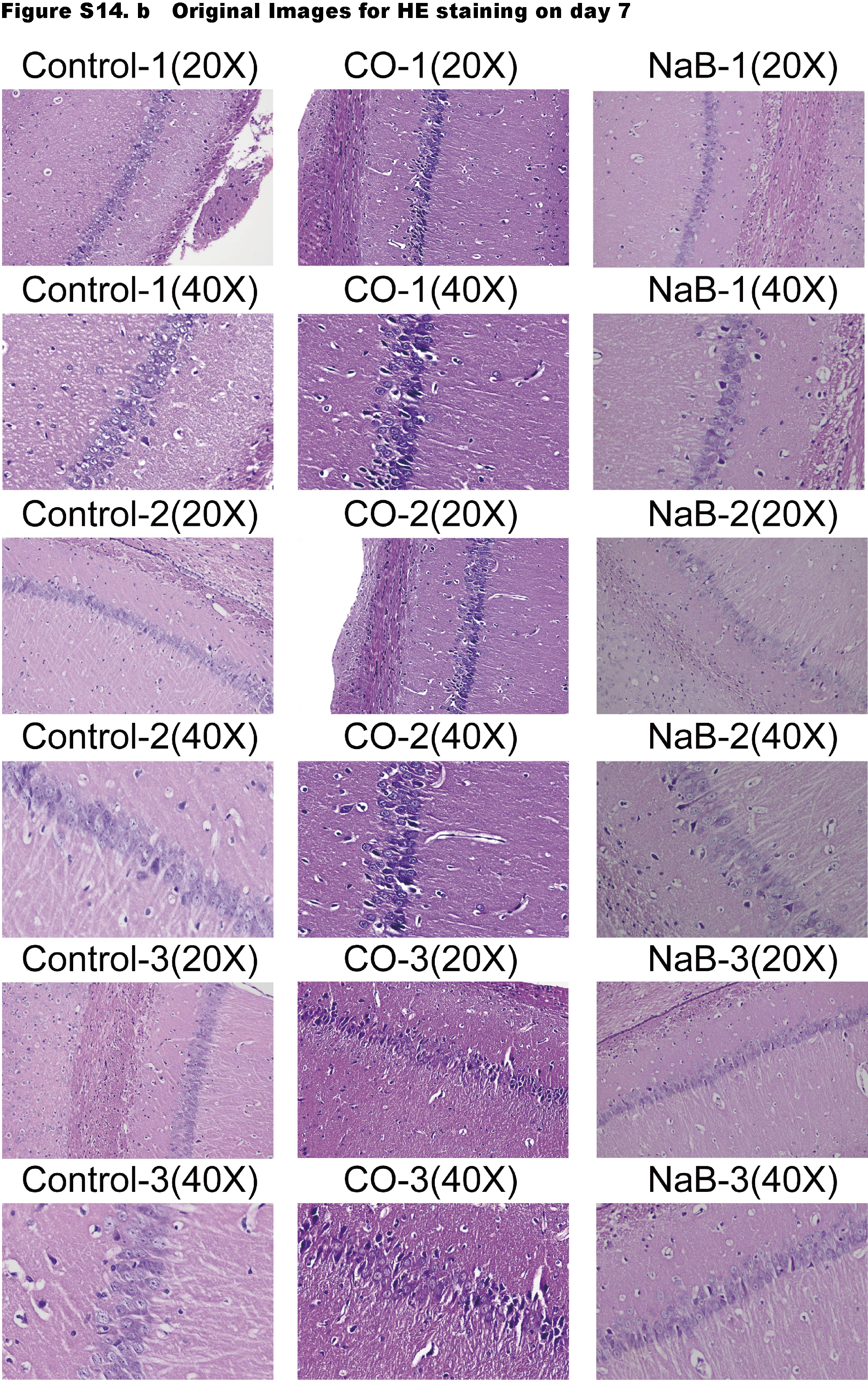

Supplement: Supplementary file 19 — Supplementary Figure S14b. [file 41598_2024_55198_MOESM19_ESM.tif]

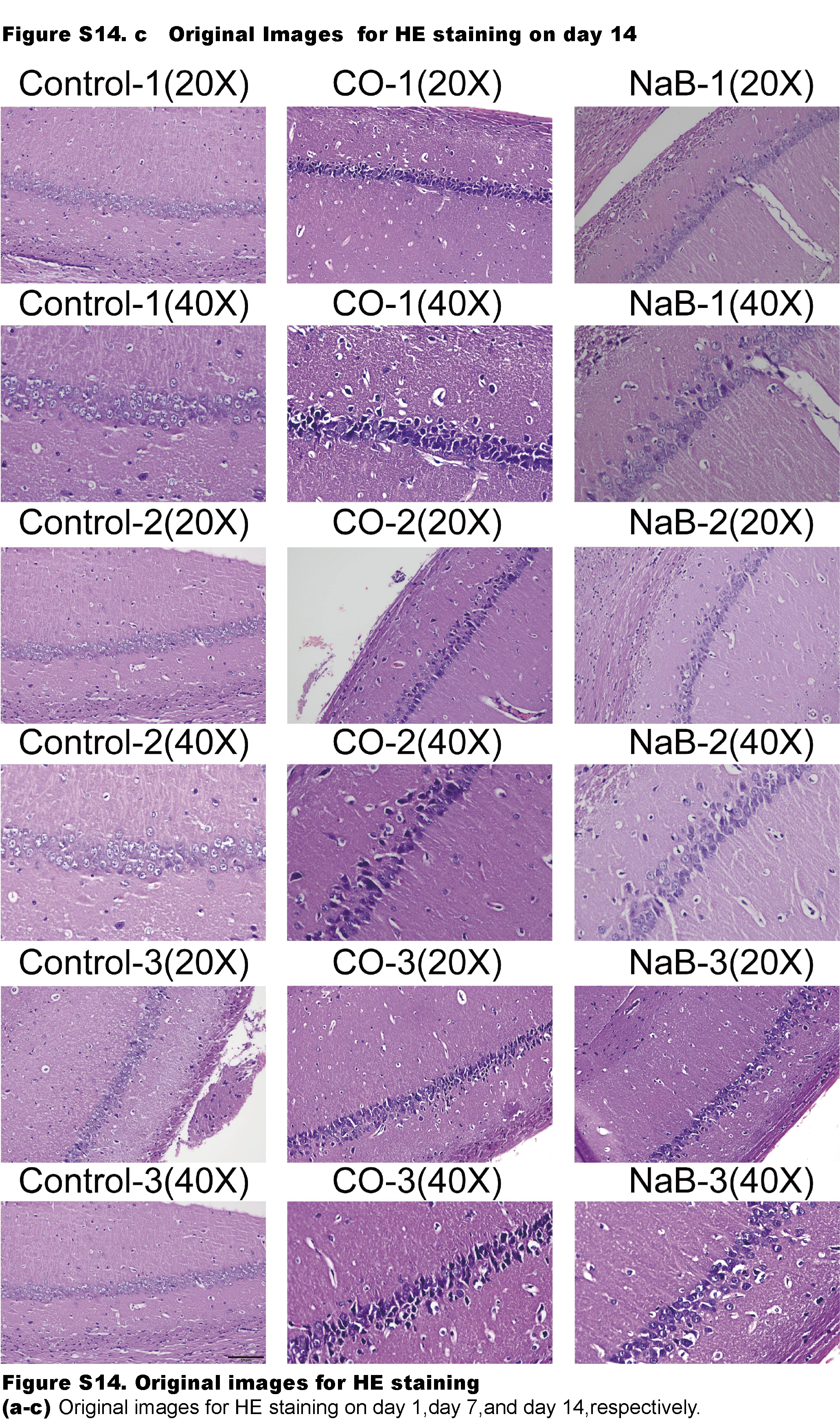

Supplement: Supplementary file 20 — Supplementary Figure S14c. [file 41598_2024_55198_MOESM20_ESM.tif]

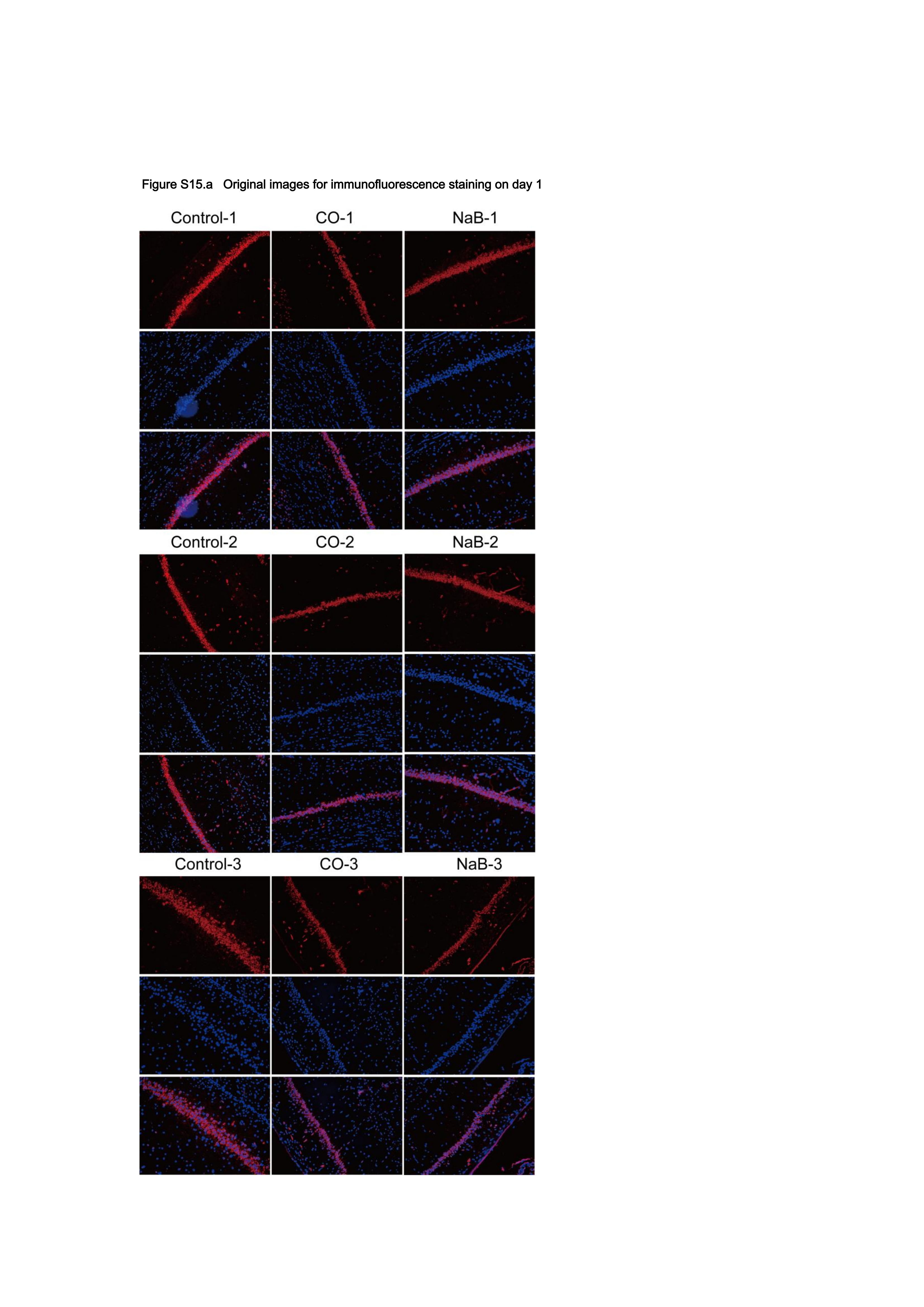

Supplement: Supplementary file 21 — Supplementary Figure S15a. [file 41598_2024_55198_MOESM21_ESM.tif]

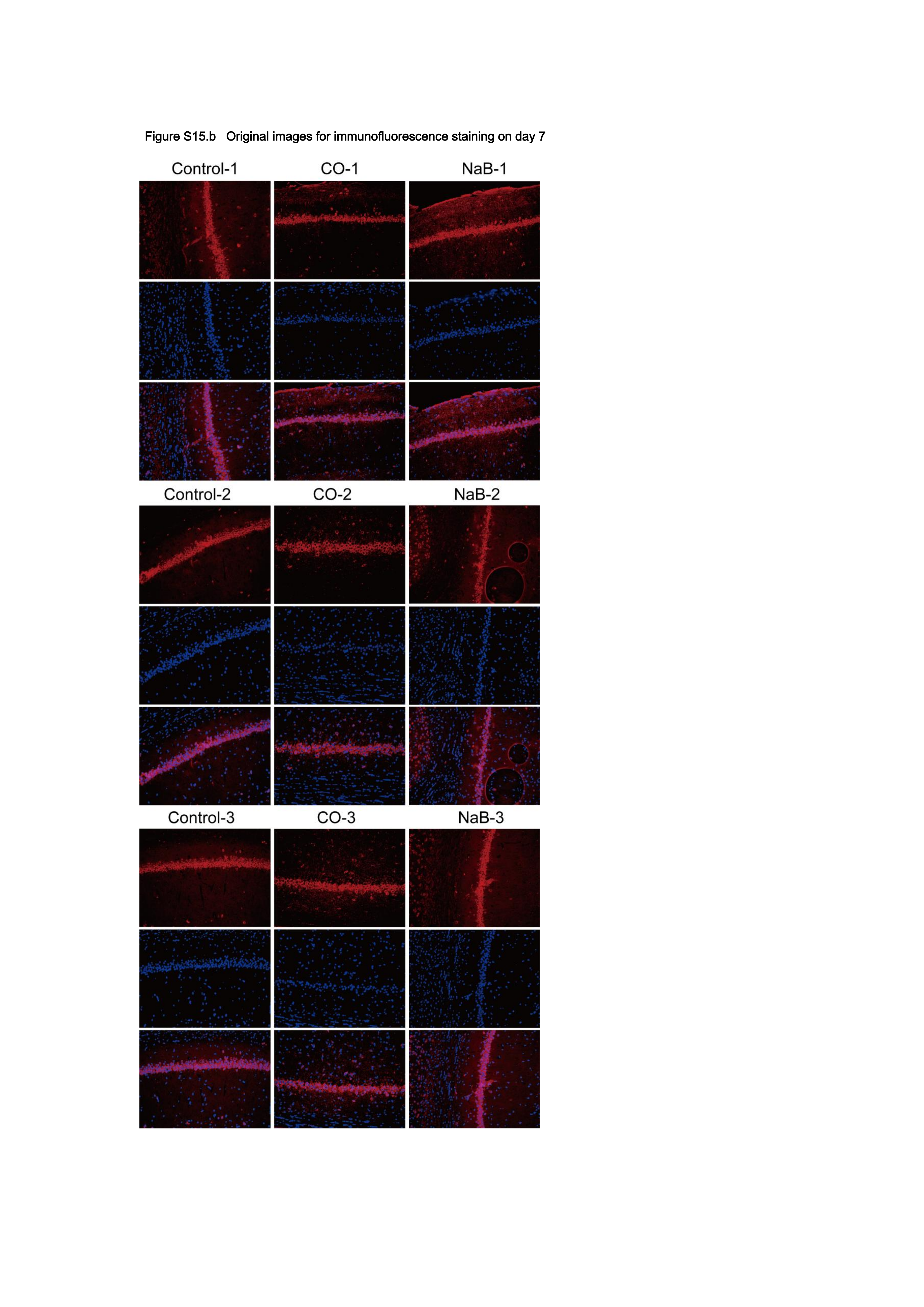

Supplement: Supplementary file 22 — Supplementary Figure S15b. [file 41598_2024_55198_MOESM22_ESM.tif]

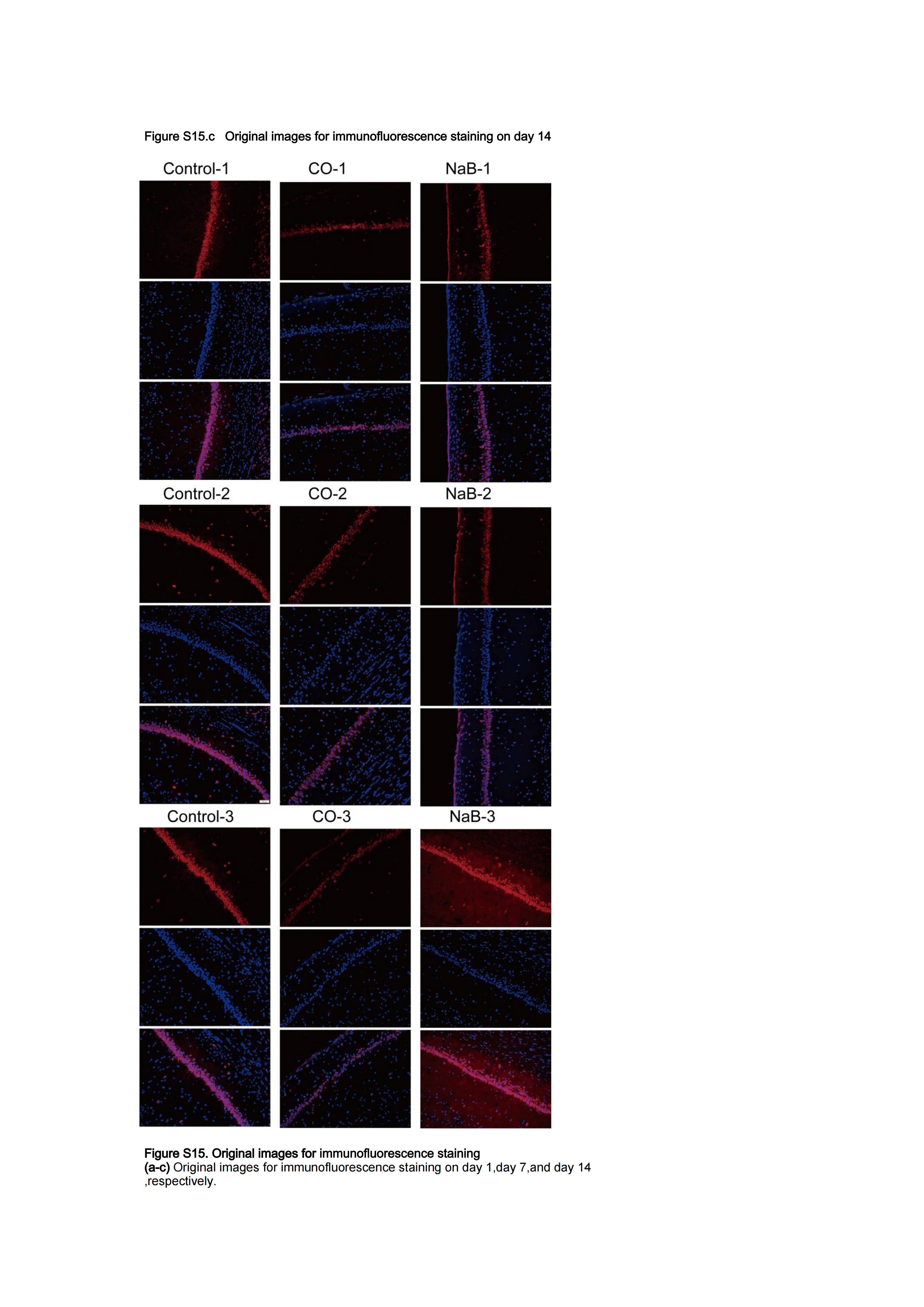

Supplement: Supplementary file 23 — Supplementary Figure S15c. [file 41598_2024_55198_MOESM23_ESM.tif]

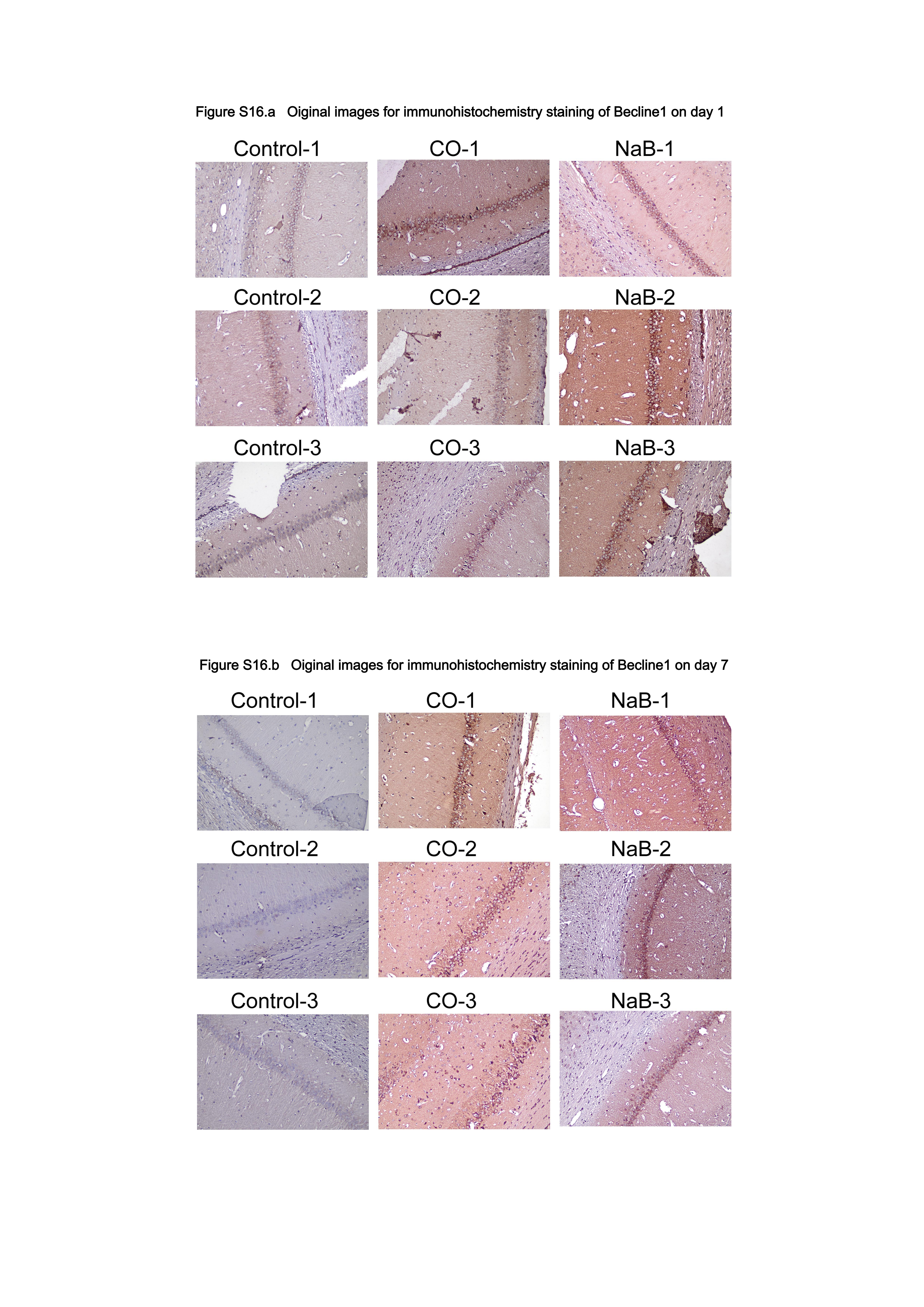

Supplement: Supplementary file 24 — Supplementary Figure S16a–b. [file 41598_2024_55198_MOESM24_ESM.tif]

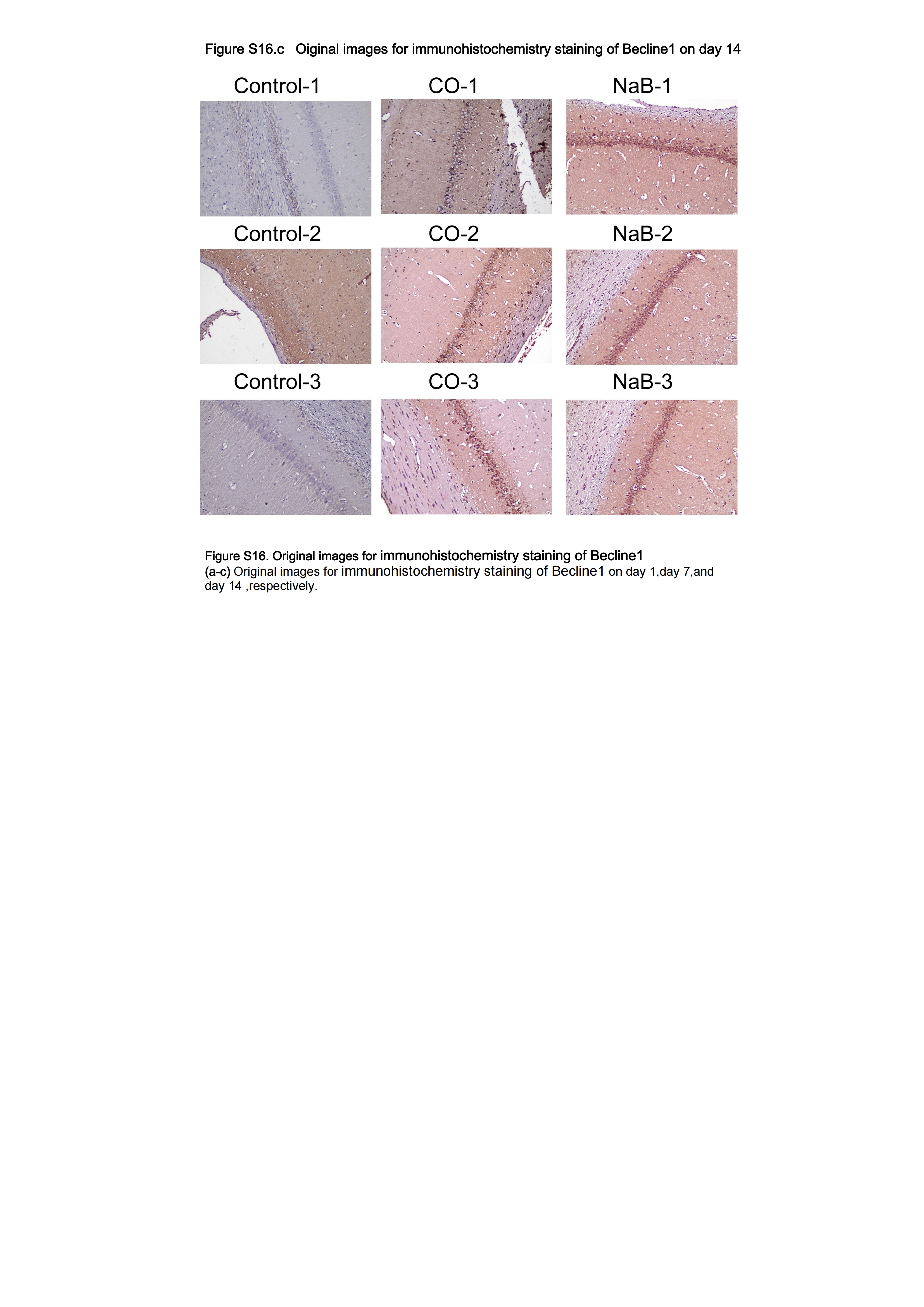

Supplement: Supplementary file 25 — Supplementary Figure S16c. [file 41598_2024_55198_MOESM25_ESM.tif]

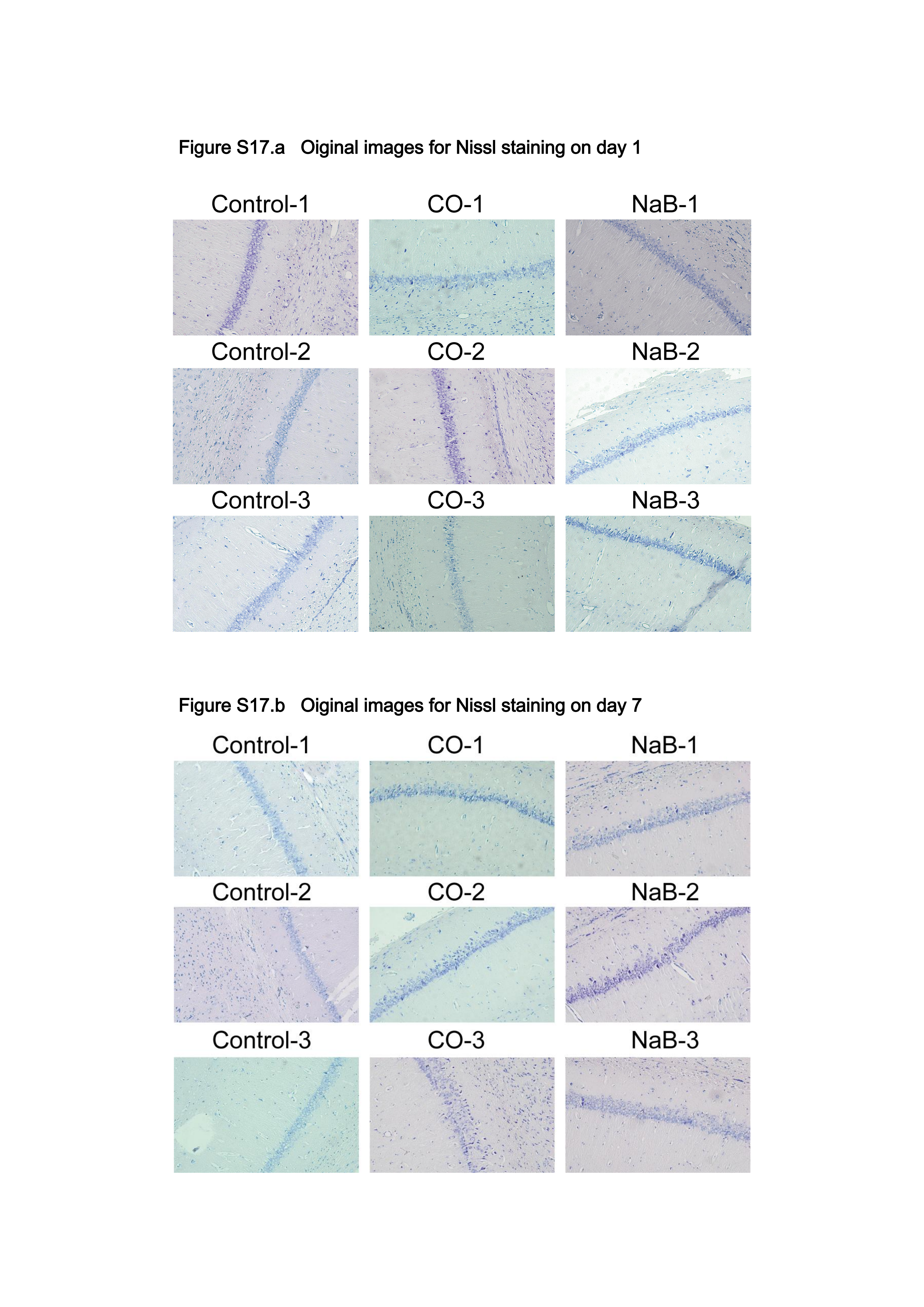

Supplement: Supplementary file 26 — Supplementary Figure S17a–b. [file 41598_2024_55198_MOESM26_ESM.tif]

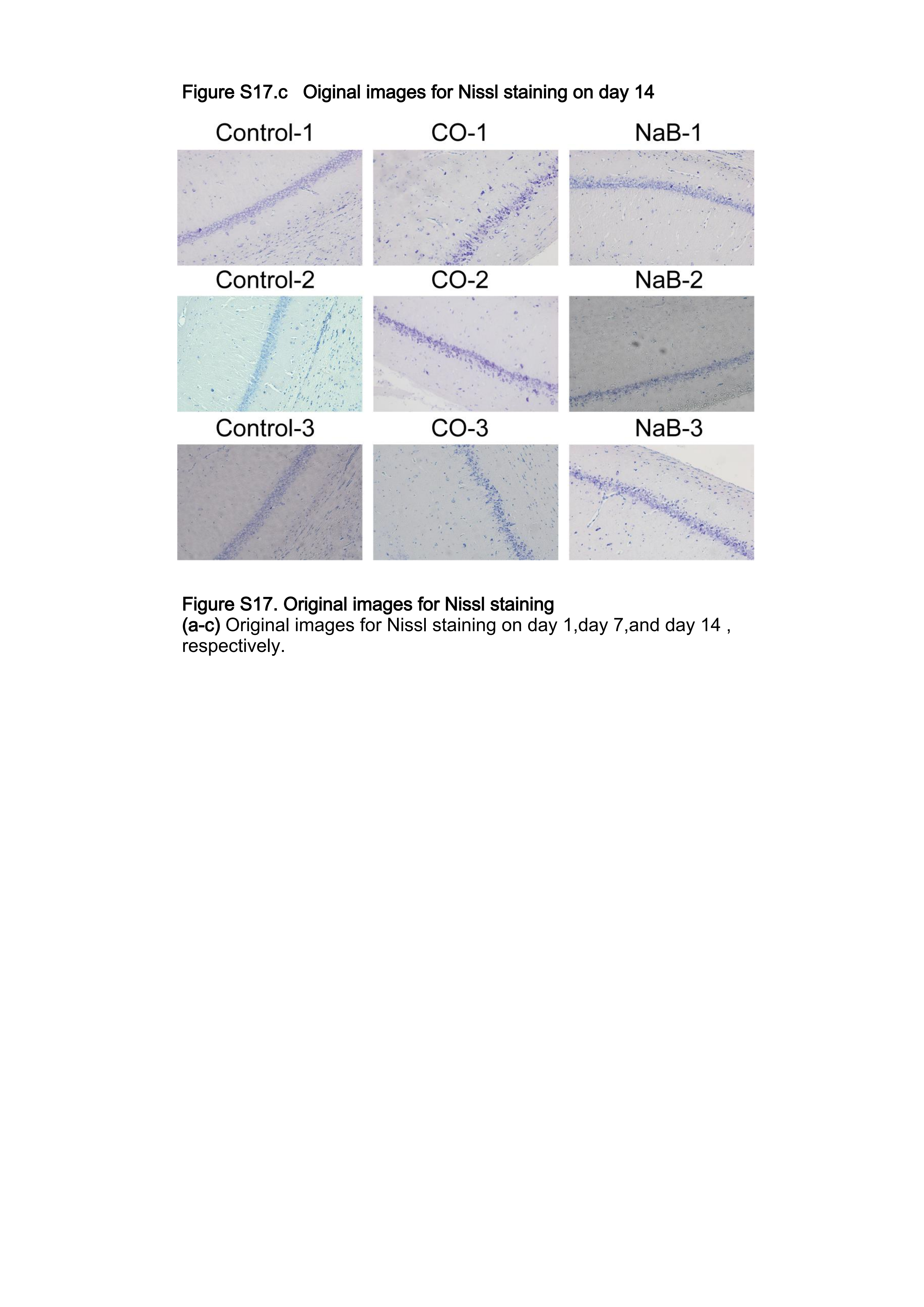

Supplement: Supplementary file 27 — Supplementary Figure S17c. [file 41598_2024_55198_MOESM27_ESM.tif]

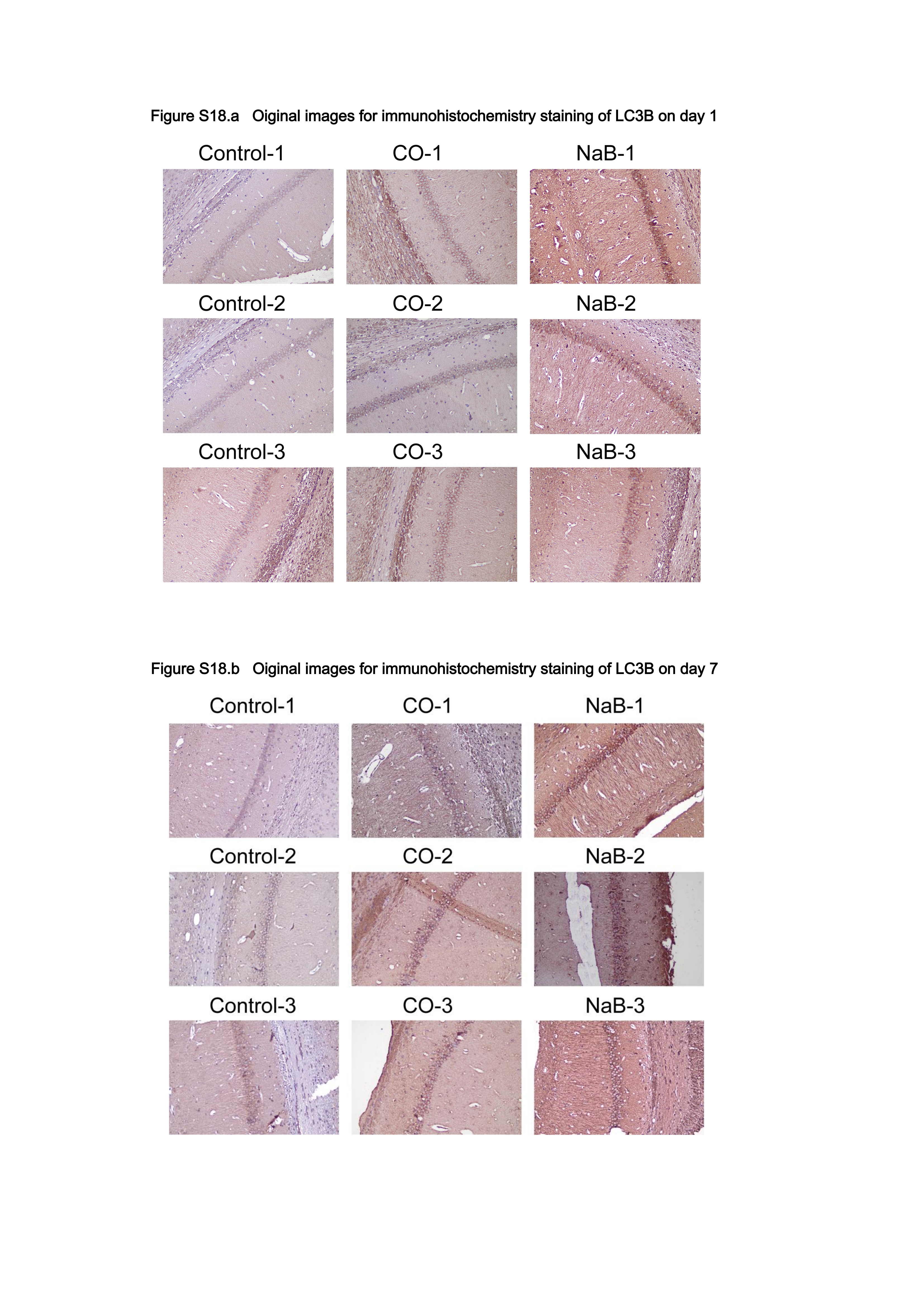

Supplement: Supplementary file 28 — Supplementary Figure S18a–b. [file 41598_2024_55198_MOESM28_ESM.tif]

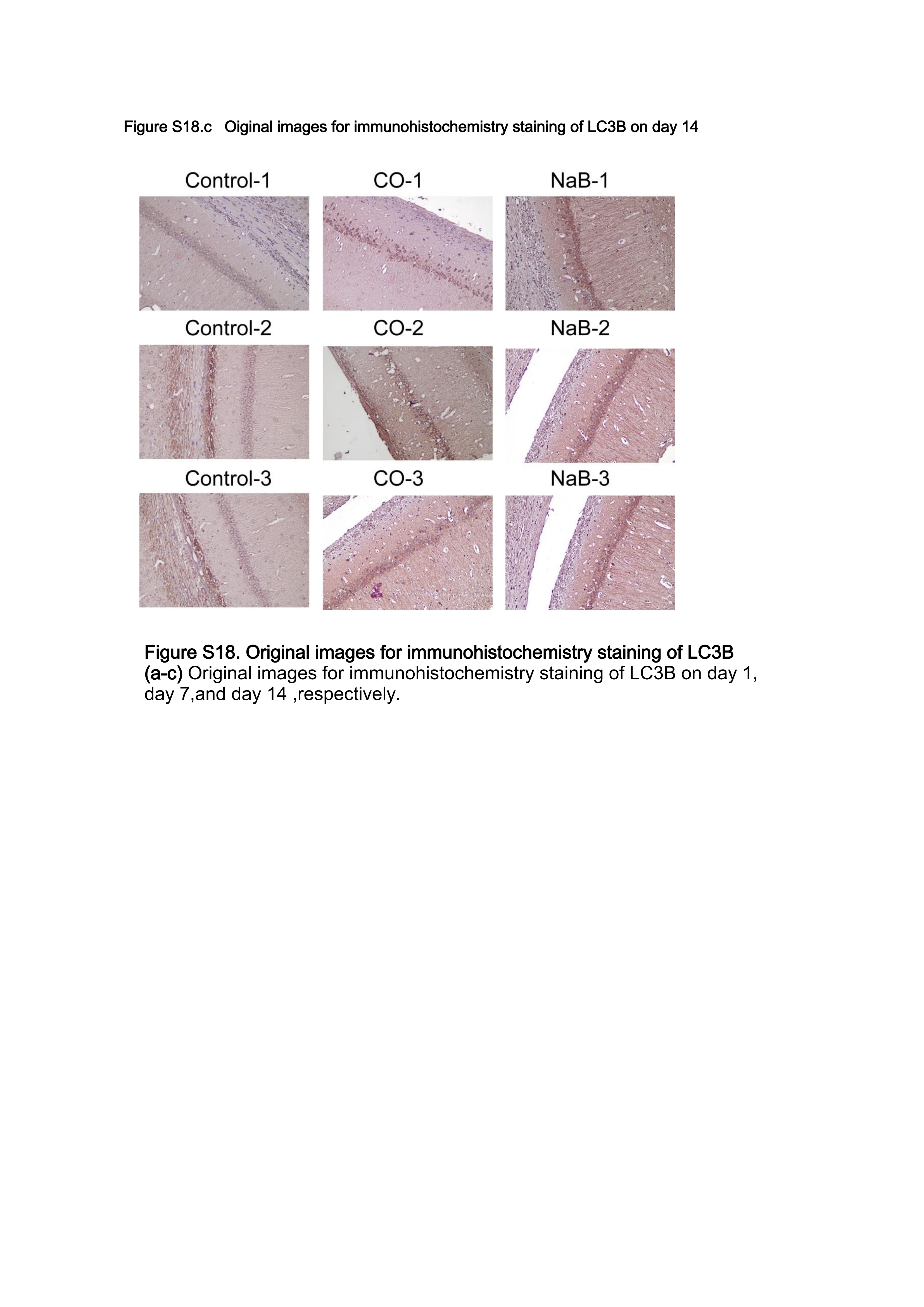

Supplement: Supplementary file 29 — Supplementary Figure S18c. [file 41598_2024_55198_MOESM29_ESM.tif]

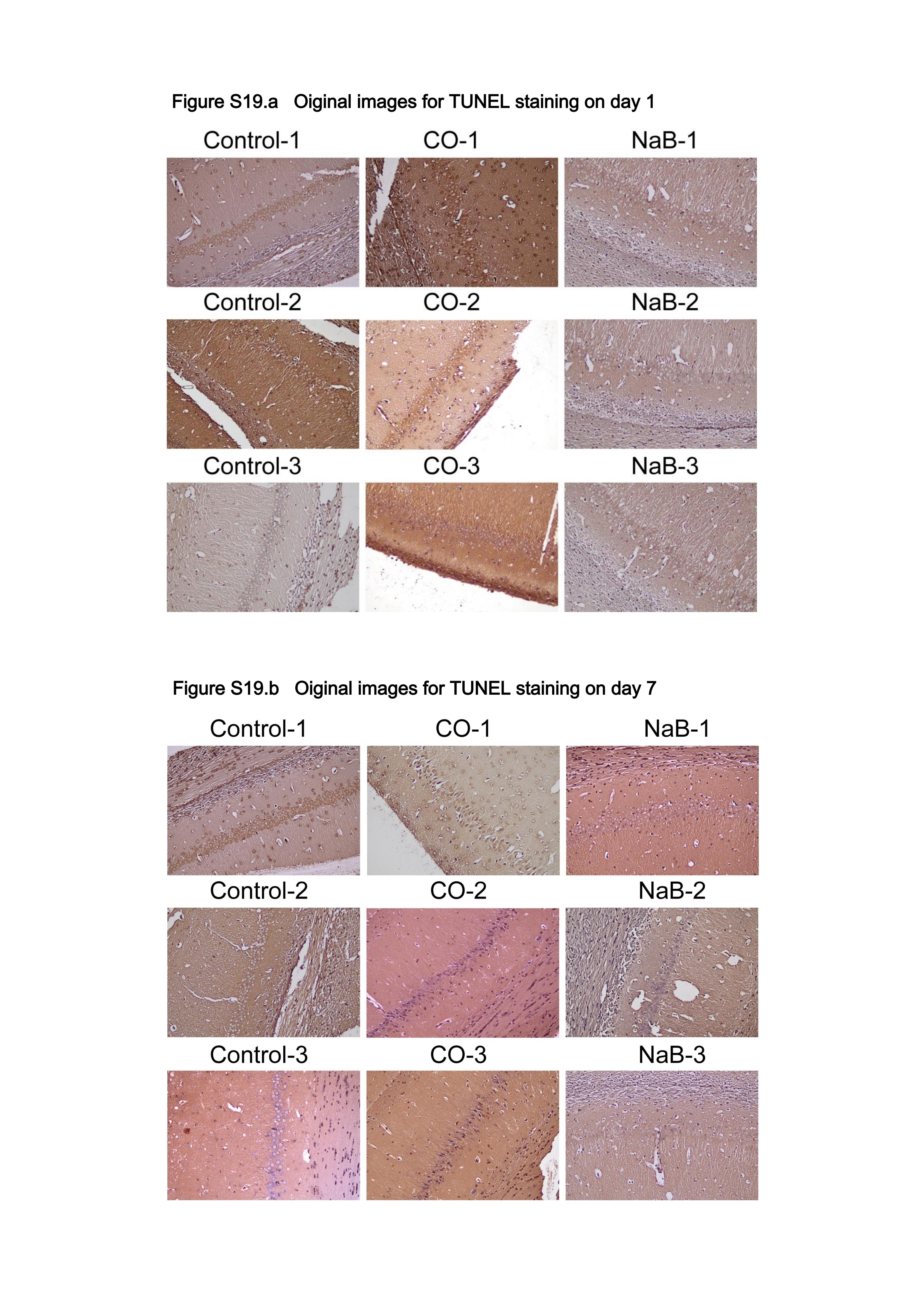

Supplement: Supplementary file 30 — Supplementary Figure S19a–b. [file 41598_2024_55198_MOESM30_ESM.tif]

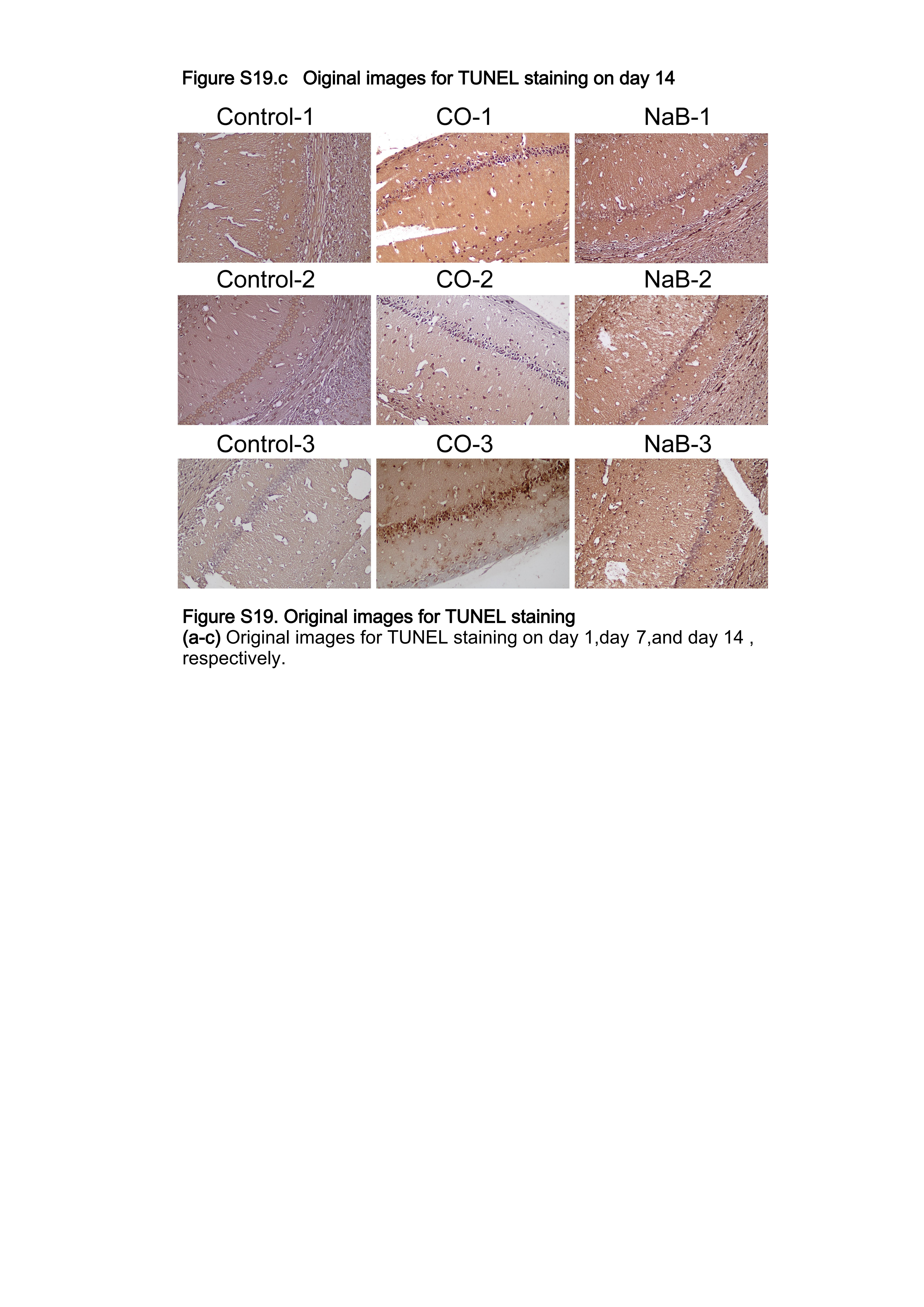

Supplement: Supplementary file 31 — Supplementary Figure S19c. [file 41598_2024_55198_MOESM31_ESM.tif]

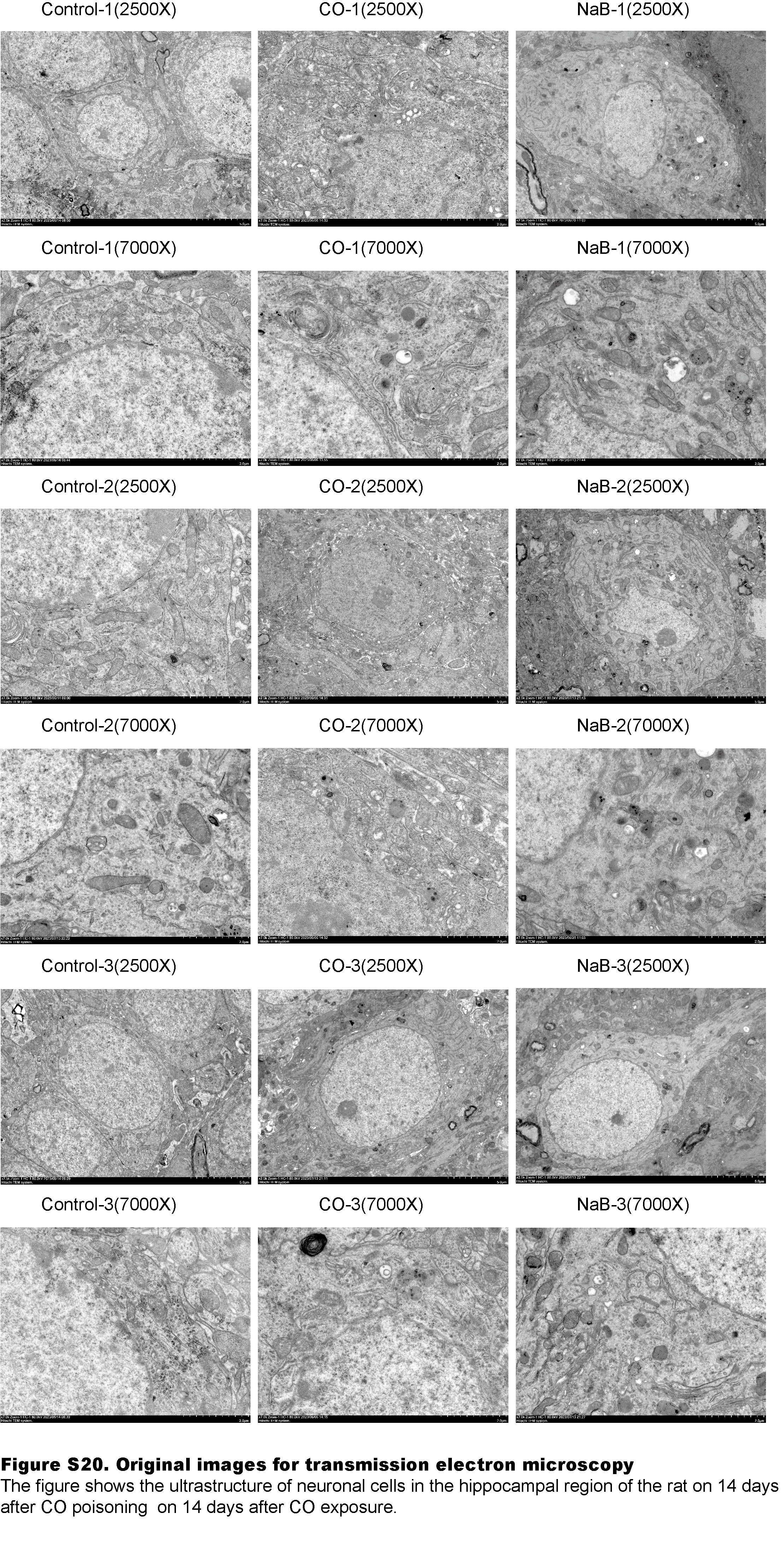

Supplement: Supplementary file 32 — Supplementary Figure S20. [file 41598_2024_55198_MOESM32_ESM.tif]
